# Maternal amoxicillin affects piglets colon microbiota: microbial ecology and metabolomics in a gut model

**DOI:** 10.1007/s00253-022-12223-3

**Published:** 2022-10-14

**Authors:** Lorenzo Nissen, Camilla Aniballi, Flavia Casciano, Alberto Elmi, Domenico Ventrella, Augusta Zannoni, Andrea Gianotti, Maria Laura Bacci

**Affiliations:** 1 Department of Agricultural and Food Sciences (DISTAL), Alma Mater Studiorum University of Bologna: Universita Di Bologna, P.za Goidanich 60, 47521 Cesena, Italy; 2 Interdepartmental Centre of Agri-Food Industrial Research (CIRI-AGRO), Alma Mater Studiorum University of Bologna: Universita Di Bologna, Via Q. Bucci 336, 47521 Cesena, Italy; 3 Department of Veterinary Medical Sciences, Alma Mater Studiorum University of Bologna: Universita Di Bologna, via Tolara di Sopra 50, 40064 Ozzano dell’Emilia (BO), Italy; 4 Health Sciences and Technologies-Interdepartmental Center for Industrial Research (CIRI-SDV), Alma Mater Studiorum University of Bologna: Universita Di Bologna, 40126 Bologna, Italy

**Keywords:** Antibiotic transfer, Antibiotic resistance, Swine reproduction, Volatilome, Microbiota, In vitro gut model

## Abstract

**Abstract:**

The first weeks of life represent a crucial stage for microbial colonization of the piglets’ gastrointestinal tract. Newborns’ microbiota is unstable and easily subject to changes under stimuli or insults. Nonetheless, the administration of antibiotics to the sow is still considered as common practice in intensive farming for pathological conditions in the postpartum. Therefore, transfer of antibiotic residues through milk may occurs, affecting the piglets’ colon microbiota. In this study, we aimed to extend the knowledge on antibiotic transfer through milk, employing an in vitro dedicated piglet colon model (MICODE—Multi Unit In vitro Colon Model). The authors’ focus was set on the shifts of the piglets’ microbiota composition microbiomics (16S r-DNA MiSeq and qPCR—quantitative polymerase chain reaction) and on the production of microbial metabolites (SPME GC/MS—solid phase micro-extraction gas chromatography/mass spectrometry) in response to milk with different concentrations of amoxicillin. The results showed an effective influence of amoxicillin in piglets’ microbiota and metabolites production; however, without altering the overall biodiversity. The scenario is that of a limitation of pathogens and opportunistic taxa, e.g., *Staphylococcaceae* and *Enterobacteriaceae*, but also a limitation of commensal dominant *Lactobacillaceae*, a reduction in commensal *Ruminococcaceae* and a depletion in beneficial *Bifidobactericeae*. Lastly, an incremental growth of resistant species, such as *Enterococcaceae* or *Clostridiaceae*, was observed. To the authors’ knowledge, this study is the first evaluating the impact of antibiotic residues towards the piglets’ colon microbiota in an in vitro model, opening the way to include such approach in a pipeline of experiments where a reduced number of animals for testing is employed.

**Key points:**

*• Piglet colon model to study antibiotic transfer through milk.*

*• MICODE resulted a robust and versatile in vitro gut model.*

*• Towards the “3Rs” Principles to replace, reduce and refine the use of animals used for scientific purposes (Directive 2010/63/UE).*

**Graphical abstract:**

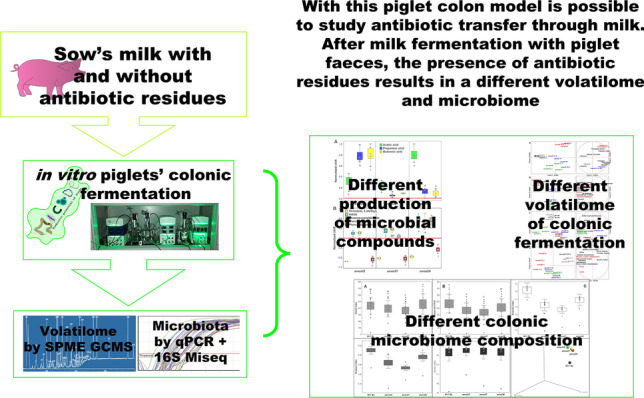

**Supplementary Information:**

The online version contains supplementary material available at 10.1007/s00253-022-12223-3.

## Introduction

In the last decades, the swine has been acknowledged as one of the most important preclinical species for a wide variety of physiological patterns. Indeed, the swine species show close similarities with humans, and the employment of pigs in research trials seems to be more widely accepted by society in terms of ethical values (Ventrella et al. 2021).

One of the latest interesting applications of this model is the study of transport of endogenous and exogenous molecules, such as pharmacological compounds, during the lactation stage which is possible via passive or active transport mechanisms, since the endothelium does not constitute a major barrier to solute movement between blood and the interstitium (Shennan and Peaker 2000). Transcellular transport requires solutes to cross the epithelial cell membranes, whereas paracellular transfer occurs between cells via leaky tight junctions (Shennan and Peaker 2000; Nauwelaerts et al. 2021). In particular, pharmacological compounds can reside in one (or more) milk fractions such as casein, fat globules, or free in the aqueous acid whey; it was acknowledged that hydrophilic drugs accumulate in the liquid medium (Ozdemir et al. 2018).

Since 2019, the European project entitled ConcePTION (n.d.) aims at generating accurate knowledge about the use of medication during pregnancy and breastfeeding (https://www.imi-conception.eu) by means of different approaches. Out of the latter, in vitro, in vivo, and in silico porcine trials have been established to generate data comparable and, most importantly translatable, to humans (Ventrella et al. 2019). Within said project, amoxicillin was chosen as the first test molecule since it is widely used for therapeutic purposes in both human and porcine medicine, with well-defined pharmacokinetics/pharmacodynamics (PK/PD) background data (Burch and Sperling 2018).

Amoxicillin is a bactericidal antibiotic in the group of aminopenicillins. When given orally to juvenile, but yet not suckling pigs, the bioavailability of amoxicillin varies between 25 and 31%, and thus, substantial drug quantities may have a direct impact on the gut microbiota (Burch and Sperling 2018). Indeed, swine gastrointestinal tract hosts a complex community of microorganisms, which compose the microbiota and take active part in immunity, digestive physiology, and nutrients metabolism (Luo et al. 2022). The microbiota of newborns is mainly transferred from the sow at birth and then later from the sow’s colostrum and milk, but it is also shaped by the surrounding environment (Isaacson and Kim 2012; Luo et al. 2022). The microbiota of piglets is dominated by *Firmicutes*, and in particular by the orders *Lactobacillales* (Petri et al. 2010) and *Clostridiales* (Yang et al. 2021). The piglet’s colon microbiota is inherited from the sow, not solely through milk, and among *Lactobacillales*, *Lactobacillaceae* early establish an important symbiosis that sculpture the intestinal epithelium up to the adult phase and bestow to the most beneficial effects derived from the microbiota (Petri et al. 2010).

In such regard, this study aims to evaluate the effect of sows’ milk with different concentrations of amoxicillin, widely used as antibiotic in piggeries, on the perturbations of the newborns gut microbiota using an innovative in vitro colon model. We used multi-unit in vitro colon model: MICODE (Nissen et al. 2021a, b) modified by using piglets’ feces from four healthy animals for a short-term colonic fermentation protocol (24 h) of different sow’s milk containing different concentrations of amoxicillin residues in comparison to a sow’s milk with no antibiotic and to a blank control. This system permitted to resemble in vitro the in vivo conditions of piglets’ gut ecology, in line with the international call to reduce animal testing (Directive 2010/63/EU; Regulation (EU) 2019/1010). In particular, it serves to highlight the shift that happens in the core microbiota and in the related volatilome after colonic fermentation. The results were obtained coupling microbiomics (qPCR and 16S-rDNA MiSeq) and metabolomics (SPME GC–MS) and studying several ecological indicators either related to microbes and molecules, as follows: (i) microbial biodiversity, (ii) microbial eubiosis, (iii) shifts in the core microbiota at high or low taxonomical levels of selected opportunistic and beneficial commensals taxa, (iv) production of postbiotics, (v) production of detrimental compounds.

## Materials and methods

### Preparative

Conventional pregnant sows were purchased from a local farm, SUIMAX di Massimo Ferri (Via San Michele 718, Valsamoggia 40,056 BO, Italy), chosen of the basis of the microbiological status of the facility and for the reproductive track records. All piglets included in the study were born from the abovementioned sows in the experimental facility of the ANFI-ASA Unit, Department of Veterinary Medical Sciences, Alma Mater Studiorum—University of Bologna (via Tolara di Sopra 50, Ozzano dell’Emilia 40,064 BO, Italy). Sows were transferred to the experimental facility 1 month prior the expected delivery date and moved to the farrowing pen one week before. Animals were fed a standard pellet formula specifically made for breeding animals, produced by a local vendor (Molini Popolari Riuniti, Ellera-Umbertide 6019 PG, Italy). Drinking water was provided ad libitum, while the daily feed ratio was divided into two portions: early morning and afternoon. Light/dark cycle was set at 12/12 h with a min of 40 lx during light hours. Temperature was set at 21 ± 1 °C to meet sows thermal needs. With regards to piglet, two heat lamps were placed in dedicated areas of the farrowing crate to reach 32 ± 1 °C. For this study, only animals previously enrolled in an experimental protocol approved by the Local Ethics Committee and the Italian Ministry of Health were used (Legislative Decree 26/2014, authorization n° 32/2021-PR, protocol number 2216A.17). The abovementioned experimental protocol already included samplings on sows and piglets.

Briefly, fecal samples from piglets were collected, processed and used as the representation of the piglets’ colon microbiota to undergo colonic short-term in vitro batch fermentation of sows’ milk with different residues of amoxicillin in comparison to another antibiotic free milk sample.

#### Piglets’ fecal samples

Fecal samples were obtained from four 7-day-old piglets, maintained refrigerated, and processed within few hours. The fecal slurry was prepared by homogenizing 8 g of pooled feces (2 g of each piglet) in 72 mL of pre-reduced phosphate-buffered saline (PBS).

#### Sows milk (treatment and control samples)

Amoxicillin (Clamoxyl® RTU, Pfizer, New York, NY) was administered to sows, SID (standardized ileal digestible) at 7 mg/kg IM (intramuscular) from the second week of lactation until weaning (day 28). Milk samplings were manually obtained at different timepoints, after a prior administration of exogenous oxytocin, and immediately frozen (− 80 °C) to preserve amoxicillin and its metabolites. Three kinds of milk employed in the in vitro fermentation experiments were obtained from two pluriparous conventional adult sows aged two and three years approximately. Amox07 and amox08 are milk samplings from the first sow, with different concentrations of amoxicillin, collected 24 h and 2 h post intramuscular administration respectively, 9 days from the onset of lactation. Amox02 is the milk sample with no amoxicillin residues from the second lactating sow, used as the positive control and collected 6 days post-parturition. The blank control was instead used as a negative control. Milk samples were stored at − 80 °C and analyzed at the bioanalytical laboratory of BioNotus® (Niel, Belgium) using a validated liquid chromatography–mass spectrometry (LC–MS/MS) method (BioNotus Method: MT-500A). Analyses were performed using Shimadzu Nexera X2 UHPLC, coupled with Shimadzu LC–MS 8050 system (Shimadzu, Kyoto, Japan). The data was acquired and processed via LabSolutions version 6.81 software (Shimadzu). The lower and upper limit of quantification of amoxicillin were 10 ng/mL and 10,000 ng/mL respectively.

#### Fecal batch-culture fermentation and sample collection

Colonic fermentations were conducted for 24 h in independent vessels on 1% (w/v) of amox02, on 1% (w/v) of amox07, on 1% (w/v) of amox08 (positive control), and on a blank control (BC) (negative control), using the in vitro gut model MICODE, obtained by the assembly of Minibio Reactors (Applikon Biotechnology BV, Delft, NL) and controlled by Lucullus PIMS software (Applikon Biotechnology BV, NL) (Nissen et al. 2021a, b). The preparation of the experiments was made according to published procedures (Koutsos et al. 2017; Wang et al. 2020; Nissen et al. 2021a, b; Venardou et al. 2021). In details, bioreactors were autoclaved at 121 °C and 100 kPa for 15 min and once cooled aseptically filled with 90 mL of anaerobic pre-sterilized fermentation medium (FM) (Venardou et al. 2021). FM contained (per liter): 5 g/L yeast extract, 10 g/L ascorbic acid, 10 g/L sodium acetate, 5 g/L (NH_4_)_2_SO_4_, 2 g/L urea, 0.2 g/L MgSO_4_7H_2_O, 0.01 g/L FeSO_4_7H_2_O, 0.007 g/L MnSO_4_xH_2_O, 0.01 g/L NaCl, 1 ml/L Tween 80, 0.05 g/L hemin, and 0.5 g/L L-cysteine hydrochloride. The pH was adjusted to 7.0. Fermentation vessels were filled aseptically with 90 mL of FM and the bioreactor headplates were mounted, including previously sterilized and calibrated sensors, i.e., pH and DO_2_ (dissolved oxygen) sensors (AppliSense, Applikon Biotechnology BV, NL). Anaerobic condition (0.0–0.1% w/v of DO_2_) in each bioreactor was obtained in about 30 min flushing with filtered O_2_-free N_2_ through the mounted-in sparger of Minibio reactors (Applikon Biotechnology BV, NL), and was constantly kept over the experiment. Temperature was set at 39 °C and stirring at 100 rpm, while pH was adjusted to 7.0 and kept throughout the experiment with the automatic addition of filtered NaOH or HCI (0.5 M). Once the exact environmental settings were reached, each of the four vessels was aseptically injected with 10 mL of pooled fecal slurry (10% w/v of pooled piglets’ feces to a final concentration of 1%, w/v) and then three of them independently with 1 mL of amox02, amox07, or amox08 (to a final concentration of 1%, w/v), while the fourth vessel was set as blank control (BC, basal medium and 1% fecal slurry only). Batch cultures were run under these controlled conditions for a period of 26.10 h during which samples were collected at 3 time points (BL, baseline; T1 = 18 h; and EP = 24 h). Baseline (BL) was defined on the first pH changes (Venema 2015) detected by Lucullus (1 read/10 s) via the pH Sensors of MICODE (AppliSense Sensors, Applikon Biotechnology BV, NL). For this work, the BL was set after 2.10 ± 0.28 h. Sampling was performed with a dedicated double syringe–filtered system (Applikon Biotechnology BV, NL) connected to a float drawing from the bottom of the vessels without perturbing or interacting with the bioreactor’s ecosystem. To guarantee a close control, monitoring, and recording of fermentation parameters, the software Lucullus 3.1 (PIMS, Applikon Biotechnology BV, Delft, NL) was used. This also allowed to keep the stability of all settings during the experiment. Fermentations were conducted in duplicate independent experiments, using for the first the fresh pooled slurry in pre-reduced PBS and for the second the same pooled slurry in pre-reduced PBS and 15% glycerol, previously stored at − 80 °C for a week (Asare et al. 2021).

#### Experimental set up and pipeline of activities

Parallel and independent vessels for amox02, amox07, amox08, and blank control were run for 24 h after the adaptation of the fecal inoculum, defined as the baseline (BL). The entire experiment consisted of 9 duplicated biological cases (*n* = 18), including 4 theses (amox02, amox07, amox08, and BC) and 3 time points (BL = 2.10 h, T1 = 18 h, and EP = 24 h) in duplicate. Samples of the different time points were used for qPCR and SPME GC–MS analyses. Pooled samples at the BL and the EPs of the 4 fermentation theses were used for 16S-rDNA MiSeq analyses (Illumina Inc, San Diego, CA, USA). After sterile sampling of 6 mL of bioreactor contents, samples were centrifuged at 16,000 × g for 7 min to separate the pellets and the supernatants, which were used for bacterial DNA extraction and SPME–GC–MS analysis, respectively (Nissen et al. 2021a, b). Specifically, microbial DNA extraction was conducted just after sampling so as not to reduce *Firmicutes* content (Nissen et al. 2021a, b). DNA samples and solid phase micro-extraction (SPME) GC–MS samples were then stored at − 80 °C. Technical replicates of analyses were conducted in duplicate for SPME GC–MS (*n* = 36), in triplicate for qPCR (*n* = 54), and in single pooled cases (*n* = 5) for MiSeq.

### Microbiomics

#### DNA extraction

Bacterial DNA was extracted from the MICODE eluates at each time points, just after sampling; at the baseline (BL, when the fecal inoculum adapted to the in vitro condition), at the intermediate time point (T1, after 18 h), and at the endpoint (EP, after 24 h) using the Purelink Microbiome DNA Purification Kit (Invitrogen, Thermo Fisher Scientific, Carlsbad, CA, USA). Bacterial DNA was extracted also from frozen sow’s milk using the NucleoSpin Food DNA Isolation Kit (Macherey–Nagel, Duren, De). Nucleic acid purity and concentration was tested on BioDrop Spectrophotometer (Biochrom Ltd., Cambridge, UK).

#### DNA amplification and sequencing by Illumina MiSeq

Samples from the BL and the EP were used for MiSeq sequencing (Illumina Inc, USA). Bacterial diversity was obtained by the library preparation and sequencing of the 16S rRNA gene. The following two amplification steps were performed: an initial PCR amplification using 16S locus-specific PCR primers (16S-341F 5′-CCTACGGGNGGCWGCAG-3′ and 16S-805R 5′-GACTACHVGGGTATCTAATCC-3′) and a subsequent amplification integrating relevant flow-cell-binding domains (5′-TCGTCG GCAGCGTCAGATGTGTATAAGAGACAG-3′ for the forward primer and 5′-GTCTCGTGGGCTCGGAGATGTGTATAAGAGACAG-3′ for the reverse overhang), and lastly unique indices selected among those available Nextera XT Index Kits were combined according to manufacturer’s instructions (Illumina Inc, USA). Both input and final libraries were quantified by Qubit 2.0 Fluorometer (Invitrogen, USA). In addition, libraries were quality-tested by Agilent 2100 Bioanalyzer High Sensitivity DNA assay (Agilent technologies, Santa Clara, CA, USA). Libraries were sequenced in a MiSeq (Illumina Inc, USA) in the paired end with 300-bp read length (Marino et al. 2019). Sequencing was conducted by IGA Technology Service Srl (Udine, Italy).

#### Sequence data analysis

Reads were de-multiplexed based on Illumina indexing system, as described in Marino et al. (2019). Sequences were analyzed using QIIME 2.0 (Caporaso et al. 2010). After filtering based on read quality and length (minimum quality = 25 and minimum length = 200), operational taxonomic units (OTUs) defined by a 97% of similarity were picked using the Uclust v1.2.22 q method (Edgar 2010), and the representative sequences were submitted to the RDP classifier (Wang et al. 2007) to obtain the taxonomy assignment and the relative abundance of each OTU using the Greengenes 16S rRNA gene database (Version 2013_8) (McDonald et al. 2012). Alpha and beta diversity analyses were performed using QIIME 2.0.

#### Absolute enumeration of bacterial groups by qPCR

Enumeration of bacterial groups was made by qPCR to quantify the microbiota at the BL and evidence changes after fermentation (Tanner et al. 2014; Westfall et al. 2018; Tsitko et al. 2019; Tamargo et al. 2022) and from the milk samples to quantify the bacterial loads, following previous protocols (Modesto et al. 2011; Nissen et al. 2021a, b). For milk samples, 8 bacterial taxa were analyzed, namely *Eubacteria*, *Firmicutes, Lactobacillales*, *Bifidobacteriaceae*, *Enterobacteriaceae*, *Clostridium* group I, *Clostridium* group IV, and *Escherichia coli.* For colonic fermentation samples, the previous 8 and other 6 taxa were analyzed, namely *Bacteroidetes*, *Bacteroides-Prevotella-Porphyromonas* (BPP) group, *Atopobium-Collinsella-Eggerthella* (ATOP) group, *Bifidobacterium longum*, *Faecalibacterium prausnitzii*, and *Akkermansia muciniphila*) (Supplemental Table S1) were assessed by qPCR on a QuantStudio 5 System (Applied Biosystem, Thermo Fisher, USA).

### Metabolomics

#### Volatilome analysis

Volatile organic compound (VOCs) evaluation was carried out on an Agilent 7890A Gas Chromatograph (Agilent Technologies, Santa Clara, CA, USA) coupled to an Agilent Technologies 5975 mass spectrometer operating in the electron impact mode (ionization voltage of 70 eV) equipped with a Chrompack CP-Wax 52 CB capillary column (50 m length, 0.32 mm ID) (Chrompack, Middelburg, the Netherlands). The SPME GC–MS protocol and the identification of volatile compounds were done according to previous reports, with minor modifications (Guerzoni et al. 2007; Di Cagno et al. 2011; Casciano et al. 2021; Nissen et al. 2021a). Briefly, 3 mL of vessel content were centrifuged at 16,000 × g for 7 min at 4 °C and then the supernatant placed into 10-mL glass vials containing 10 μL of the internal standard (4-methyl-2-pentanol) to a final concentration of 4 mg/L. Samples were then equilibrated for 10 min at 45 °C. SPME fiber, coated with carboxen-polydimethylsiloxane (85 μm), was exposed to each sample for 40 min. Preconditioning, absorption, and desorption phases of SPME–GC analysis, and all data-processing procedures were carried out according to previous publications (Di Cagno et al. 2011; Casciano et al. 2021; Nissen et al. 2021a). Briefly, before each head space sampling, the fiber was exposed to the GC inlet for 10 min for thermal desorption at 250 °C in a blank sample. The samples were then equilibrated for 10 min at 40 °C. The SPME fiber was exposed to each sample for 40 min, and finally the fiber was inserted into the injection port of the GC for a 10-min sample desorption. The temperature program was 50 °C for 1 min, then programmed at 1.5 °C/min to 65 °C, and finally at 3.5 °C/min to 220 °C, which was maintained for 25 min. Injector, interface, and ion source temperatures were 250, 250, and 230 °C, respectively. Injections were carried out in split-less mode and helium (3 mL/min) was used as a carrier gas. Identification of molecules was carried out by searching mass spectra in the available databases (NIST 11 MSMS library and the NIST MS Search program 2.0 (NIST, Gaithersburg, MD, USA). Each VOC was relatively quantified in percentage (limit of detection, LOD = 0.001 mg/kg) (Bonfrate et al. 2020).

#### Shift of main microbial VOCs

In samples prior to in vitro colonic fermentation (BL) (Supplemental Table S2), the main microbial metabolites related to fermentation of foods were also absolutely quantified in mg/kg with the aforementioned SPME GC–MS approach and the internal standard, but with different cutoffs: LOQ (limit of quantification) = 0.03 mg/kg and LOD = 0.01 mg/kg) (Di Cagno et al. 2011; Casciano et al. 2021; Nissen et al. 2021a). For these compounds, samples at T1 and EP were compared to the BL and values were expressed as shifts. Values were computed as follows: (i) each single compound was normalized (mean centering method) within its dataset, which included cases from amox02, amox07, and amox08, and the blank control at different time points; (ii) the BL dataset (Supplemental Table S2) was then subtracted to the fermentation time points; (iii) post hoc analysis was done to compare the sample productions of a single molecule.

### Data processing and statistical analysis

For metabolomics, one-way ANOVA model (*p* < 0.05) was used to determine significant VOCs among the raw data of peak’s area of the GC–MS chromatograms. The significant VOCs (*n* = 65) representing the total volatilome of the experiments were analyzed differently: (i) 8 main VOCs related to microbial fermentation of foods were absolutely quantified and normalized and their BL values were subtracted from T1 and EP values and represented as blox plots, including post hoc Tukey HSD test (*p* < 0.05); (ii) the remaining volatilome was relatively quantified, sorted for main chemical classes and super-normalized, then each dataset was computed for principal component analysis (PCA) to distribute the results on a plane and coupled to multivariate ANOVA (MANOVA) (*p* < 0.01) to address specific contributes by categorical predictors.

For the sequencing data analysis, the QIIME pipeline version 2.0 was used. Within-community diversity (alpha diversity) was calculated using observed OTUs, Chao1 Shannon, Simpson, and Good’s coverage indexes with 10 sampling repetitions at each sampling depth. Student’s *t*-test was applied to compare the latest sequence/sample values of different treatments within an index. Analysis of similarity (ANOSIM) and the ADONIS test were used to determine statistical differences between samples (beta diversity) following the QIIME compare_categories.py script and using weighted and unweighted phylogenetic UniFrac distance matrices. Principal coordinate analysis (PCoA) plots were generated using the QIIME beta diversity plots workflow (Marino et al. 2019).

For microbiomics, ANOVA model for group comparison (BL versus EPs) (*p* < 0.05) was performed for MiSeq and MANOVA (*p* < 0.05) model (categorized for the time points and the treatments) was performed for qPCR. Afterwards, the significant variables and others of peculiar interest were selected and the shifts in abundance were calculated as Log_2_(F/C) (Love et al. 2014). Then, post hoc Tukey HSD test on the raw data (*p* < 0.05) was performed to define differences among treatments (MiSeq and qPCR) or time points (qPCR). The baselines of values for the volatilome and for the microbiota were that obtained sampling just after adaptation of the microbiota to the bioreactor condition (Nissen et al. 2021b). Normalization of datasets was performed with the mean centering method. Statistics and graphics were made with Statistica v.8.0 (Tibco, Palo Alto, CA, USA).

The NCBI Bioproject PRJNA862673 is available at https://www.ncbi.nlm.nih.gov/bioproject/862673 including Biosamples and relative SRAs, which will be release at least 2022–12-15, or with the release of linked data, whichever is first.

## Results

### Amoxicillin LC–MS/MS quantifications

Amox07 milk sample was collected 24 h post administration; amoxicillin was found below limit of quantification (i.e., < 10 ng/mL). Instead, amox08, collected 2 h post maternal administration, was quantified as 32.741 ng amoxicillin/mL. Amox02 was not analyzed as the sow was never treated with amoxicillin; this sample was used as positive control.

### Microbiomics

#### Analysis of the biodiversity in the microbiota by relative quantification of 16S-rDNA

The microbiota diversity indices were analyzed both to study the impact of different amoxicillin residues in the sow’s milk on microbial population of piglets’ colon and to assess population’s stability during fermentation of the different bioreactors (Supplemental Fig. S1). The BL value (as defined by first pH decrease) was compared to the EPs of fermentation of different treatments. Considering richness, it is unquestionable that an increase (observed OTUs) cannot happen during in vitro fermentation (Isenring et al. 2021), and reductions in respect to the BL were significantly different just for EPs of amox02 and amox07. A reduction in abundance index (Chao 1) from the BL to the EP was recorded for amox02 and amox07, while amox08 scored a slight increase, although significant differences were just that of the highest values (amox08) in respect to the lowest values (amox07). Significant reduction in evenness (Shannon) from the BL to the EP were seen for any substrate, and different values were recorded at the EP of amox08 in respect to the lowest values of amox07. This latter feature could be a first clue to a possible perturbation of microbiota eubiosis. Reductions in dominance (Simpson) were seen from the BL to the EP for any substrate, but significantly just for amox07. This latter feature could be ascribed to the reduction at the EP of a dominant phylum. Additionally, the Good’s index, relative to rare OTUs, was kept similar from the BL to the EPs of any milk substrate with just slight reductions, but no significative differences. This feature means that the stability of MICODE environment was maintained throughout the entire experimental period, because the rare taxa, which need strict ecological conditions, were still present at the EPs. When the bacterial diversity between samples (beta diversity) was examined with Bray–Curtis analysis, the pooled sample relative to the BL was set not so much distant, although discriminated in respect to the samples at the EP of fermentation, as demonstrated by principal coordinate analysis (PCoA) based on an unweighted (qualitative) phylogenetic UniFrac distance matrix.

#### Analysis of the shift in the phyla of microbiota by relative quantification of 16S-rDNA

Results from microbiota analyses at the phylum level (Table 1) have defined that the core microbiota of any sample was ruled by two main phyla with relative abundance higher than 10%, and three minors with relative abundance lower than 10%. *Firmicutes* and *Bacteroidetes*, accounted for almost the 80% of the whole pie, while *Actinobacteria*, *Proteobacteria*, and *Fusobacteria* accounted for the remaining. In any fermentation sample, *Firmicutes* and *Bacteroidetes* were reduced in respect to the BL, although not significantly. *Actinobacteria* were reduced significantly in any milk fermentations, while *Fusobacteria* and *Proteobacteria* were increased, but significantly just for the latter. The unaffected changes of the core microbiota make us generally believe that an equilibrium among such wide taxa was maintained even after fermentation.

**Table 1:** Shifts of the microbiota at the phylum level from 16S-rDNA sequencing

#OTU ID	% R.Q	Log_2_(F/C)			ANOVA*
	Baseline	amox02	amox07	amox08	*p* value
*Euryarchaeota*	0.01^a^	− 2.50^b^	− 3.03^b^	− 2.56^b^	0.001626
*Bacteria*; Other	0.04^a^	− 2.00^b^	− 1.99^b^	− 1.35^b^	0.017493
*Actinobacteria*	2.71^a^	− 2.95^b^	− 2.64^b^	− 1.59^b^	0.024128
*Bacteroidetes*	21.66	− 1.18	− 1.08	− 2.56	0.080744
*Firmicutes*	61.69	− 0.81	− 0.41	− 0.32	0.173732
*Fusobacteria*	8.12^a^	2.09^b^	1.91^b^	1.99^b^	0.009156
*Proteobacteria*	5.75	1.83	1.11	1.24	0.189654
*Synergistetes*	0.02^a^	− 3.76^b^	0.00^a^	− 3.24^b^	0.004404

^*^One-way ANOVA with *p* < 0.05. *R.Q.*, relative quantity. ^abc^Letters indicate significant differences within a line by Tukey’s honestly significant differences (HSD) test (*p* < 0.05)

#### Analysis of the shift in the families of microbiota by relative quantification of 16S-rDNA

Results from the microbiota analysis at the family level (Table 2) evidenced a scenario discriminated by the fermentation and seldom by the severity of amoxicillin concentration.

**Table 2:** Shifts of the microbiota at the family level from 16S-rDNA sequencing

#OTU ID	% R.Q	Log_2_(F/C)			ANOVA*
	M11 BL	amox02	amox07	amox08	*p* value
*Actinomycetaceae*	2.55^a^	− 3.11^b^	− 2.61^b^	− 1.61^b^	0.024667
*Bifidobacteriaceae*	0.06 ^a^	− 2.34^b^	− 3.10^b^	− 3.89^b^	0.001018
*Bacteroidaceae*	18.48	− 0.99	− 0.88	− 2.41	0.120363
*Porphyromonadaceae*	1.30^a^	− 3.04^b^	− 3.11^b^	− 4.22^b^	0.002328
*Prevotellaceae*	0.83^a^	− 5.71^b^	− 5.24^b^	− 5.85^b^	0.000033
*Rikenellaceae*	0.26^a^	− 7.31^b^	− 7.84^b^	− 6.78^b^	0.000008
*Sphingobacteriaceae*	0.72^a^	− 3.74^b^	− 3.86^b^	− 2.91^b^	0.002006
*Staphylococcaceae*	0.01^b^	1.34^a^	1.19^a^	0.42^ab^	0.000149
*Enterococcaceae*	0.17^b^	5.48^a^	5.86^b^	6.43^b^	0.027770
*Lactobacillaceae*	34.52^a^	− 1.89^b^	− 3.14^b^	− 2.19^b^	0.010708
*Streptococcaceae*	0.93^a^	− 2.08^b^	− 2.45^b^	− 1.51^b^	0.017605
*Clostridiales*; other	0.09^a^	− 3.73^b^	− 5.26^b^	− 4.79^b^	0.000991
*Clostridiaceae*	2.44	1.84	3.52	2.21	0.057749
*Lachnospiraceae*	9.30^a^	− 2.18^b^	− 2.59^b^	− 2.29^b^	0.001559
*Peptococcaceae*	0.66	− 0.53	− 0.84	− 0.60	0.096887
*Peptostreptococcaceae*	2.20^a^	− 0.75^b^	− 1.55^c^	0.25^b^	0.000012
*Ruminococcaceae*	7.04^a^	− 2.73^b^	− 1.37^b^	− 2.93^b^	0.002811
*Veillonellaceae*	2.13	1.19	− 1.85	1.91	0.638170
*Coriobacteriaceae*	0.34	− 1.02	0.61	0.66	0.805605
*Coprobacillaceae*	0.26^b^	0.00^b^	− 0.05^b^	1.68^a^	0.000092
*Erysipelotrichaceae*	1.52	− 1.84	− 2.51	− 1.76	0.009737
*Fusobacteriaceae*	8.12^a^	2.09^b^	1.91^b^	1.99^b^	0.009156
*Alcaligenaceae*	0.26^a^	− 0.39^a^	− 2.12^b^	− 1.53^b^	0.045315
*Desulfovibrionaceae*	0.66	− 4.72	− 5.25	− 5.17	0.000032
*Campylobacteraceae*	0.01	1.83	2.17	1.50	0.113515
*Enterobacteriaceae*	4.40	2.15	1.38	1.19	0.256852
*Pasteurellaceae*	0.08^b^	3.01^a^	3.42^a^	5.46^a^	0.023245

^*^One-way ANOVA with *p* < 0.05. *R.Q.*, relative quantity. ^abc^Letters indicate significant differences within a line by Tukey’s honestly significant differences (HSD) test (*p* < 0.05)

Indeed, amox08 during colonic fermentation was able to reduce the content of opportunistic *Porhyromonadaceae* and limit the growth of *Staphylococcaceae*, *Enterobacteriaceae*, and *Desulfovibrionaceae* in a significative difference in respect to the milk control with no antibiotic residues (amox02). Oppositely, the antibiotic residues exerted an undesired effect towards important beneficial taxa of the piglets’ colon microbiota, due to a wider range of targets. This effect was different in respect to the different capacity of a taxon to generically resist to insults. In particular, this effect was dramatically high in sensitive *Bifidobacteriaceae*, which were almost depleted after amox08 fermentation, and in sensitive *Ruminococcaceae*, which were reduced of almost three-folds, in respect to the BL and two time more than the milk without antibiotic residues. Also, this effect was observed in dominant *Lactobacillaceae*. Unexpectedly, this effect was observed also for important commensal fibrolytic bacteria, such as *Bacteroidaceae*, that was reduced of 2.4-folds in respect to the BL, although not significantly. Furthermore, it is observed in some taxa a competitive advantage by the presence of antibiotic residues, recording an increased abundance. This phenomenon was particularly strong in those bacterial taxa phenotypically heterogeneous. For example, from the superior taxonomic level of *Lactobacillales*, two family behaved oppositely; as we have just said, the *Lactobacillaceae* were reduced (from 34.5% at the baseline to 7.6% at the endpoint of fermentation with amox08), but the *Enterococcaceae* were fostered (from 0.16% at the baseline to 14.5% at the endpoint of fermentation with amox08). Similarly, from the superior level of *Gammaproteobacteria*, the *Enterobacteriaceae* were more limited (from 4.4% at the baseline to 19.5% and 10.0% at the endpoint of amox02 and amox08 fermentations, respectively), but the *Pasteurellaceae* were increased (from 0.07% at the baseline to 0.6% and 3.3% at the endpoint of amox02 and amox08 fermentations, respectively). We can summarize the presence of antibiotic residues in the milk can modulate the microbiota of piglets via four main actions. (i) A desired inhibitory effect towards several opportunistic bacterial taxa; (ii) an inhibitory effect towards sensitive commensal taxa; (iii) a stimulation of tough (generally resistant to stress) bacterial taxa.

#### Analysis of the shift in the genera and species of microbiota by relative quantification of 16S-rDNA

In order to try to account the shift previously observed to some specific taxa, a relative quantification of 16S-rDNA was performed (Table 3). Specifically, the reduction of *Lactobacillaceae* in contrast to the increase in *Enterococcaceae* has been generated by some key players, as *Lactobacillus crispatus* (from 9.7% at the baseline to 2.2% and 1.0% at the endpoint of amox02 and amox08 fermentations, respectively), *Lactobacillus antrii* (from 8.3% at the baseline to 3.7% and 3.6% at the endpoint of amox02 and amox08 fermentations, respectively), *Lactobacillus gasseri* (from 9.5% at the baseline to 1.6% and 1.4% at the endpoint of amox02 and amox08 fermentations, respectively), and *Lactobacillus delbruecki* (from 1.5% at the baseline to 0.3% and 0.1% at the endpoint of amox02 and amox08 fermentations, respectively). Oppositely, under the *Enterococcaceae*, the species that were overrepresented were *Enterococcus durans* (from 0.1% at the baseline to 3.2% and 4.5% at the endpoint of amox02 and amox08 fermentations, respectively) and *Enterococcus faecalis* (from cutoff levels at the baseline to 3.7% and 9.0% at the endpoint of amox02 and amox08 fermentations, respectively).

**Table 3:** Shifts of the microbiota at the genus and species level from 16S-rDNA sequencing

#OTU ID	% R.Q	Log_2_(F/C)			ANOVA*
	Baseline	amox02	amox07	amox08	*p* value
*Methanobrevibacter*	0.01^a^	− 2.50^b^	− 3.03^b^	− 2.56^b^	0.001626
*Actinomyces*	2.54^a^	− 3.10^b^	− 2.60^b^	− 1.61^b^	0.024542
*Corynebacterium*	0.02	− 0.68	− 2.21	− 0.24	0.360138
*Bifidobacterium*	0.06^a^	− 3.34^b^	− 3.10^b^	− 3.89^b^	0.001018
*Bacteroides*	18.48^a^	− 0.99^b^	− 0.88^ab^	− 2.41^c^	0.017239
*Porphyromonas*	0.09^a^	− 4.50^b^	− 6.35^b^	− 5.29^b^	0.000364
*Parabacteroides*	1.21^a^	− 2.97^b^	− 3.01^b^	− 4.17^b^	0.002679
*Prevotella*	0.83^a^	− 5.71^b^	− 5.24^b^	− 5.85^b^	0.000033
*Rikenella*	0.26^a^	− 7.31^b^	− 7.84^b^	− 6.78^b^	0.000008
*Enterococcus*	0.17	5.48	5.86	6.43	0.127750
*Lactobacillus*	34.52^a^	− 1.99^b^	− 3.14^b^	− 2.19^b^	0.010708
*Streptococcus*	0.93^a^	− 2.08^b^	− 2.45^b^	− 1.51^b^	0.017605
*Clostridiaceae*; other	0.23^a^	− 3.73^b^	− 5.26^b^	− 4.79^b^	0.000991
*Clostridium*	2.07	1.88	3.52	1.74	0.434835
*Finegoldia*	0.01	6.09	8.24	7.94	0.284612
*Mogibacterium*	0.21^a^	− 4.52^b^	− 4.19^b^	− 3.29^b^	0.001492
*Lachnospiraceae*; other	2.61^a^	− 1.74^b^	− 3.10^b^	− 2.22^b^	0.017499
*Blautia*	0.03	− 1.92	− 0.59	− 0.74	0.185554
*Dorea*	1.02^a^	− 3.11^b^	− 4.92^b^	− 3.22^b^	0.003279
*Roseburia*	0.01	− 0.44	− 0.70	− 0.24	0.189321
*Ruminococcus*	5.59^a^	− 2.41^b^	− 3.38^b^	− 2.24^b^	0.007038
*Peptococcus*	0.66	− 0.54	− 0.84	− 0.60	0.056183
*Peptostreptococcaceae*; other	0.09	2.98	2.14	4.27	0.399730
*Clostridium*	0.31^a^	− 1.73^bc^	− 2.28^c^	− 1.08^b^	0.049276
*Peptostreptococcus*	1.79^a^	− 1.92^b^	− 2.73^b^	− 1.45^b^	0.026758
*Faecalibacterium*	0.47^a^	− 2.92^b^	− 3.20^b^	− 2.55^b^	0.001763
*Oscillospira*	1.77^a^	− 3.06^b^	− 3.31^b^	− 1.69^b^	0.025503
*Ruminococcus*	4.73^a^	− 4.32^b^	− 5.51^b^	− 4.12^b^	0.000510
*Megasphaera*	0.06^a^	− 3.74^b^	− 5.59^b^	− 3.12^b^	0.003442
*Negativicoccus*	1.10	2.07	− 0.97	2.83	0.522025
*Phascolarctobacterium*	0.96^a^	− 2.34^b^	− 6.12^c^	− 2.64^b^	0.016017
*Veillonella*	0.02	2.05	− 2.12	0.09	0.765288
*Atopobium*	0.02^a^	− 2.50^bc^	− 4.03^c^	− 1.75^b^	0.026410
*Collinsella*	0.01	− 1.07	− 1.82	0.15	0.530060
*Eggerthella*	0.06	− 1.13	− 0.18	0.78	0.983175
*Coprobacillus*	0.25	0.01	− 0.05	1.69	0.672057
*Bulleidia*	1.01^a^	− 1.83^b^	− 3.47^b^	− 1.66^b^	0.031552
*Eubacterium*	0.49^a^	− 1.95^b^	− 1.51^b^	− 2.18^b^	0.011357
*Fusobacterium*	8.12^a^	2.09^b^	1.91^b^	1.99^b^	0.009146
*Sutterella*	0.26	− 0.39	− 2.37	− 1.56	0.237870
*Bilophila*	0.03^a^	− 3.26^b^	− 2.21^b^	− 2.15^b^	0.008941
*Desulfovibrio*	0.63^a^	− 6.10^b^	− 5.92^b^	− 5.81^b^	0.000004
*Escherichia*	4.39	1.99	1.24	1.15	0.240319
*Aggregatibacter*	0.06	3.45	3.87	5.91	0.475903
*Pseudomonas*	0.05	− 1.34	− 0.65	− 1.47	0.091075
*Methanobrevibacter;s__smithii*	0.01^a^	− 2.50^b^	− 3.03^b^	− 2.56^b^	0.001626
*Bacteroides;s__acidifaciens*	0.04^a^	− 2.62^b^	− 3.95^b^	− 3.48^b^	0.004352
*Bacteroides;s__heparinolyticus*	0.23^a^	− 2.92^b^	− 3.23^b^	− 2.27^b^	0.004990
*Bacteroides;s__ovatus*	0.37^a^	− 2.58^b^	− 4.41^b^	− 3.45^b^	0.006093
*Bacteroides;s__pyogenes*	4.18^a^	1.00^b^	1.09^b^	− 1.72^c^	0.025060
*Bacteroides;s__uniformis*	0.20^a^	− 1.71^b^	− 2.38^b^	− 4.30^c^	0.031170
*Bacteroides;s__vulgatus*	1.26^a^	− 2.37^b^	− 2.46^b^	− 1.61^b^	0.014477
*Parabacteroides;s__distasonis*	1.12^a^	− 3.05^b^	− 3.07^b^	− 4.17^b^	0.002280
*Prevotella;s__*	0.64^a^	− 6.11^b^	− 5.93^b^	− 6.46^b^	0.000009
*Enterococcus;s__durans*	0.13^b^	4.69^a^	5.14^a^	5.13^a^	0.038408
*Enterococcus;s__faecalis*	0.03	6.84	7.18	8.13	0.220472
*Lactobacillus;s__antri*	8.33^a^	− 1.20^b^	− 1.68^b^	− 1.15^b^	0.021077
*Lactobacillus;s__crispatus*	9.75^q^	− 2.13^b^	− 5.85^c^	− 3.19^bc^	0.018745
*Lactobacillus;s__delbrueckii*	1.50^a^	− 2.07^b^	− 5.76^c^	− 3.18^bc^	0.020505
*Lactobacillus;s__gasseri*	9.54^a^	− 2.57^b^	− 4.74^b^	− 2.75^b^	0.008456
*Streptococcus;s__hyointestinalis*	0.33^a^	− 2.73^b^	− 3.1 ^b^	− 2.43^b^	0.002672
*Clostridiaceae;Other;Other*	0.14^a^	− 3.34^b^	− 2.12^b^	− 2.12^b^	0.011455
*Clostridium;s__baratii*	0.02	− 0.32	− 0.48	− 0.38	0.054278
*Clostridium;s__butyricum*	0.01	11.77	13.60	11.35	0.427891
*Clostridium;s__cadaveris*	0.01	2.85	1.56	4.72	0.059228
*Clostridium;s__frigidicarnis*	0.01^a^	− 3.63^b^	− 3.65^b^	− 2.69^b^	0.003019
*Clostridium;s__perfringens*	1.90	− 1.49	− 2.57	− 0.44	0.226359
*Finegoldia;s__magna*	0.02	4.35	6.71	6.76	0.313308
*Dorea;s__*	1.01^a^	− 3.11^b^	− 4.97^b^	− 3.22^b^	0.003398
*Roseburia;s__faecis*	0.02^a^	− 1.17^b^	− 0.70^ab^	− 1.24^b^	0.001921
*Ruminococcus;s__*	3.12^a^	− 3.28^b^	− 4.35^b^	− 3.14^b^	0.021094
*Ruminococcus;s__gnavus*	2.47^a^	− 1.76^b^	− 2.69^b^	− 1.58^b^	0.047608
*Faecalibacterium;s__*	0.45^a^	− 3.12^b^	− 3.35^b^	− 2.67^b^	0.029017
*Faecalibacterium;s__prausnitzii*	0.02^a^	− 0.91^ab^	− 1.44^b^	− 1.10^b^	0.025503
*Negativicoccus;s__succinicivorans*	1.10^b^	2.07^a^	− 0.97^c^	2.83^a^	0.015768
*Veillonella*;*other*	0.02^c^	4.24^a^	0.30^c^	1.64^b^	0.018016
*Adlercreutzia;s__*	0.17^b^	− 0.53^b^	1.38^a^	1.17^a^	0.026410
*Eggerthella;s__lenta*	0.06	− 1.13	− 0.18	0.78	0.135460
*Coprobacillus;s__cateniformis*	0.25^b^	0.02^b^	− 0.03^b^	1.69^a^	0.031552
*Bulleidia;s__*	1.01^a^	− 1.83^b^	− 3.47^c^	− 1.66^b^	0.005019
*Fusobacterium;s__gonidiaformans*	1.45^b^	4.54^a^	4.36^a^	4.45^a^	0.000031
*Sutterella;s__parvirubra*	0.02^c^	3.44^a^	1.41^b^	2.20^ab^	0.008941
*Escherichia*;*other*	4.23	1.99	1.25	1.14	0.266914
*Escherichia;s__*	0.04	1.74	1.20	0.78	0.222009
*Escherichia;s__albertii*	0.11	1.98	1.17	1.29	0.475903
*Actinobacillus;s__porcinus*	0.02^a^	− 3.05^b^	− 4.70^b^	− 4.69^b^	0.003434
*Acinetobacter;s__lwoffii*	0.19^a^	− 6.22^c^	− 6.28^c^	− 1.93^b^	0.031993

^*^One-way ANOVA with *p* < 0.05. *R.Q.*, relative quantity. ^abc^Letters indicate significant differences within a line by Tukey’s honestly significant differences (HSD) test (*p* < 0.05)

For the intestinal health of piglets, the role of *Clostridiales* is crucial, because represents a large portion of the core microbiota (Yang et al. 2021). Actually, our samples accounted for about the 24% of total microbiota at the baseline. They include some pathogen targets of amoxicillin, but others are commensals butyrate producers. For example, while the stress sensitive *Lachnospiraceae* or the *Ruminococcaceae* were around tenfold inhibited in amox08 colon-fermented microbiota in comparison to the control, with the same milk the opportunistic *Veillonaceae* and *Peptostreptococcaceae* slightly increased and even more in *Clostridiaceae* (fivefold). In particular, within this latter family, another phenotypical split had happened, indeed even if all the three major genera of this family were fostered by any milk substrate, the sole genus *Clostridium* grew less than the control (7.6% and 6.9% at the endpoint of amox08 and amox02 fermentations, respectively), while genera *Finegoldia* (0.7% and 2.5% at the endpoint of amox02 and amox08 fermentations, respectively) and *Anaerococcus* (0.3% and 1.7% at the endpoint of amox02 and amox08 fermentations, respectively) were increased much more than the control. Noteworthy, even deeper in the genus *Clostridium*, some species were limited while others were fostered after fermentation with amox08. For example, the harmful *Clostridium perfringens* (from 1.9% at the baseline to 1.4% at the endpoint), *Clostridium baratii*, and *Clostridium frigidicarnis* were underrepresented, while *Clostridium butyricum* and *Clostridium cadaveris* were overrepresented.

#### Absolute enumeration of selected taxa of milk

We firstly considered milk microbiota to give a more complete picture of all the ecological factors affecting microbial shift in MICODE gut model. For a robust description of the core microbiota and its shifts produced after fermentation of the different milk samples, we performed qPCR absolute quantifications of 10 selected targets related to healthy piglets’ colon ecology, either at top or low taxonomic levels. We have also considered the bacterial loads of 8 principal bacterial taxa common in sow’s milk. Considering milk, generally there were significant differences mainly comparing the milk samples with no antibiotic residues (amox02) or the milk samples with the lowest antibiotic residues (amox07) to the milk samples with antibiotic residues (amox08). In the milk samples, total bacterial load accounted for a mean of 1.12E + 06 cells/mL and amox08 had 44% significantly less abundance than the milk with no antibiotic residues. *Firmicutes* content had a mean value of 2.78E + 05 and amox08 had 40% significantly less abundance than amox02. *Lactobacillales* content had a mean value of 2.01E + 05 and amox08 had 40% and 33% significantly less abundance than amox02 and amox07, respectively. *Clostridium* group I and *Clostridium* group IV had means values of 1.91E + 04 and 2.59E + 04, and amox08 had 32% and 46% significantly less abundance than amox02, respectively. *Enterobacteriaceae* had a mean value of 1.41E + 04 and amox08 had 34% significantly less abundance than amox02. In this family, *Escherichia coli* was detectable just in the amox02 and amox07 samples, accounting for a mean value of 1.68E + 02. A similar outcome was also seen for the content of *Bifidobacteriaceae* that was detectable just in the amox02 and amox07 samples, accounting for a mean value of 1.8E + 04. From these results, it is possible to summarize that the presence of amoxicillin residues in the milk diminished depending on concentration its indigenouos microflora.

#### Absolute enumeration of selected taxa of colonic fermentation samples

With the same analytical approach, the shifts occurred during MICODE fermentation were considered. In general, significant differences were found for the milk substrates, but not for the blank control. At the BL, the abundance similarly averaged (no significant differences among BL raw data) for 1.05E + 10 and trended to increase, except for the blank control, with no significant differences (Table 4). Considering the two main phyla, in fermentation samples, *Firmicutes* and *Bacteroidetes* had opposite trends. The former was increased by amox02 and amox07 and reduced by amox08 (of about 2.62E + 09 cells/mL), the latter was reduced by each milk samples, but not significantly for amox02. In particular, amox08 reduced *Bacteroidetes* of about 1.39E + 09 cells/mL, which was circa 9 time more the reduction of amox02. In the taxon *Firmicutes*, the *Lactobacillales* recorded an increase for amox02 and significant reduction just for amox08, which was reduced almost thrice after fermentation and approximately 6 times more than amox07. The *Clostridium* group I was significantly reduced at the EP just by amox08 (− 2.32-folds) and significantly increased with amox02 and amox07, of 1.31- and 1.43-folds, respectively. The *Clostridium* group IV was reduced by each treatment and significantly just by amox08, but the reduction scored by amox08 was almost thrice that of amox02. In the taxon *Bacteroidetes*, the BPP group quantified mainly the *Bacteroides* abundance, and recorded significant shifts in reduction for any milk sample, with amox08 having more than the double the strength of amox02. Considering the *Enterobacteriaceae* and the *E. coli* taxa, significant reductions from the BL on were observed just for the amox08 sample at the EP. Similarly, the *Bifidobacteriaceae* were significantly reduced just by amox08, but values under the detection limit were observed for amox07. In conclusion, just amox08 fermentation was able to contain and reduce opportunistic bacteria in piglets’ colon, but also reduced the abundance of commensals and beneficials.

**Table 4:** Enumeration (cells/mL) by qPCR of core microbiota of milk and fermentation samples

Sample	Cells/mL			Log_2_(F/C)		MANOVA
	Milk ± SD*	BL raw**	BL mean ± SD	T1	EP	
*Eubacteria*						
amox02	1.80E + 06 ± 1.50E + 06^A^	1.26E + 10	1.05E + 10 ± 1.92E + 09^b^	1.08^aA^	0.26^ab^	0.014929
amox07	1.23E + 06 ± 1.12E + 06^A^	1.01E + 10	1.05E + 10 ± 1.92E + 09	0.54^AB^	0.31	0.060255
amox08	3.25E + 05 ± 1.21E + 05^B^	8.84E + 09	1.05E + 10 ± 1.92E + 09	0.19^B^	0.26	0.822842
Blank	n.a	1.06E + 10	1.05E + 10 ± 1.92E + 09	− 0.43^B^	− 0.43	0.088726
	0.046181	0.072272		0.034454	0.987142	*p* value
*Firmicutes*						
amox02	4.16E + 05 ± 2.76E + 05	1.62E + 09	2.73E + 09 ± 9.73E + 08^b^	0.72^abA^	1.20^aaA^	0.037431
amox07	3.37E + 05 ± 1.89E + 05	3.17E + 09	2.73E + 09 ± 9.73E + 08^b^	1.28^aA^	1.73^aA^	0.005016
amox08	7.98E + 04 ± 3.47E + 04	3.41E + 09	2.73E + 09 ± 9.73E + 08^a^	− 0.29^aB^	− 4.15^bC^	0.018042
Blank	n.a	2.92E + 09	2.73E + 09 ± 9.73E + 08	− 0.43^B^	0.29^B^	0.276141
	0.061105	0.076691		0.012030	0.000066	*p* value
*Bacteroidetes*						
amox02	n.a	1.37E + 09	1.68E + 09 ± 3.73E + 08	− 0.85^A^	− 0.16^A^	0.089309
amox07	n.a	1.58E + 09	1.68E + 09 ± 3.73E + 08^a^	− 1.16^bB^	− 1.44^bB^	0.002010
amox08	n.a	2.10E + 09	1.68E + 09 ± 3.73E + 08^a^	− 2.81^bB^	− 2.67^bC^	0.001914
Blank	n.a	1.58E + 09	1.68E + 09 ± 3.73E + 08^a^	− 0.43^aA^	− 1.28^bB^	0.001463
		0.970638		0.003979	0.002111	*p* value
*Lactobacillales*						
amox02	2.97E + 05 ± 2.75E + 05^A^	1.37E + 09	9.26E + 08 ± 4.18E + 07	0.08^A^	0.33^A^	0.064451
amox07	2.53E + 05 ± 1.67E + 05^A^	1.58E + 09	9.26E + 08 ± 4.18E + 07	− 0.10^A^	− 0.23^A^	0.085205
amox08	5.33E + 04 ± 2.89E + 04^B^	2.10E + 09	9.26E + 08 ± 4.18E + 07 ^a^	− 2.59^bB^	− 2.96^bB^	0.000001
Blank	n.a	1.58E + 09	9.26E + 08 ± 4.18E + 07	− 0.42^A^	− 0.34^A^	0.060350
	0.043189	0.999926		0.000002	0.000007	*p* value
*Bacteroides–Prevotella–Porphyromonas*						
amox02	n.a	5.91E + 08	5.69E + 08 ± 3.23E + 07	− 0.38^A^	− 0.68^A^	0.060603
amox07	n.a	5.42E + 08	5.69E + 08 ± 3.23E + 07^a^	− 1.26^bA^	− 1.47^bB^	0.000001
amox08	n.a	6.02E + 08	5.69E + 08 ± 3.23E + 07^a^	− 2.27^bB^	− 3.19^cC^	0.000002
Blank	n.a	5.42E + 08	5.69E + 08 ± 3.23E + 07^a^	− 0.41^aA^	− 0.81^bA^	0.000187
		0.901999		0.000615	0.000569	*p* value
*Bifidobacteriaceae*						
amox02	1.24E + 04 ± 2.67E + 02^B^	3.81E + 02	2.35E + 02 ± 1.42E + 02	0.54	0.93^A^	0.712690
amox07	2.34E + 04 ± 3.63E + 03^A^	9.68E + 01	2.35E + 02 ± 1.42E + 02	n.d	n.d	n.d
amox08	n.d	2.32E + 02	2.35E + 02 ± 1.42E + 02^a^	− 4.37^c^	− 2.65^bB^	0.039056
Blank	n.a	2.32E + 02	2.35E + 02 ± 1.42E + 02	− 0.15	− 0.13^A^	0.946939
	0.000001	0.951484		0.088766	0.036499	*p* value
*Enterobacteriaceae*						
amox02	1.80E + 04 ± 5.36E + 02^B^	1.30E + 07	1.39E + 07 ± 1.31E + 06	0.68^A^	1.28^AB^	0.301428
amox07	2.07E + 04 ± 5.42E + 03^AB^	1.25E + 07	1.39E + 07 ± 1.31E + 06^b^	0.96^aA^	2.56^aA^	0.000256
amox08	3.50E + 03 ± 2.39E + 03^A^	1.51E + 07	1.39E + 07 ± 1.31E + 06^a^	− 1.42^bB^	− 2.54^bC^	0.003090
Blank	n.a	1.50E + 07	1.39E + 07 ± 1.31E + 06	0.12^A^	0.35^B^	0.073044
	0.000001	0.907242		0.001772	0.000737	*p* value
*Clostridium* group IV						
amox02	5.07E + 04 ± 9.35E + 03	2.51E + 08	2.51E + 08 ± 1.01E + 07	− 0.55	− 0.57^A^	0.074610
amox07	1.22E + 04 ± 3.68E + 03	2.45E + 08	2.51E + 08 ± 1.01E + 07	− 0.27	− 0.30^A^	0.436952
amox08	1.50E + 04 ± 1.17E + 04	2.64E + 08	2.51E + 08 ± 1.01E + 07^a^	− 1.43^b^	− 3.02^cB^	0.000072
Blank	n.a	2.44E + 08	2.51E + 08 ± 1.01E + 07	− 0.42	− 0.27^A^	0.080431
	0.307957	0.993674		0.079424	0.000007	*p* value
*Clostridium* group I						
amox02	2.07E + 04 ± 6.10E + 03^A^	8.02E + 06	1.07E + 06 ± 5.67E + 06	1.05^A^	1.31^A^	0.003502
amox07	3.42E + 04 ± 3.15E + 03^A^	7.03E + 06	1.07E + 06 ± 5.67E + 06	1.21^A^	1.43^A^	0.004502
amox08	2.49E + 03 ± 1.16E + 03^B^	1.73E + 07	1.07E + 06 ± 5.67E + 06	− 1.30^B^	− 2.32^C^	0.046666
Blank	n.a	1.20E + 07	1.07E + 06 ± 5.67E + 06	− 0.20^A^	− 0.28^B^	0.705529
	0.000071	0.987058		0.000275	0.000003	*p* value
*Escherichia coli*						
amox02	2.03E + 02 ± 5.33E + 01^A^	1.50E + 06	1.63E + 06 ± 1.36E + 05^b^	0.51^abA^	1.48^aA^	0.000010
amox07	1.33E + 02 ± 2.64E + 01^B^	1.58E + 06	1.63E + 06 ± 1.36E + 05^b^	1.10^aA^	1.07^aA^	0.041208
amox08	n.d	1.76E + 06	1.63E + 06 ± 1.36E + 05^a^	− 1.49^bB^	− 3.50^cB^	0.000004
Blank	n.a	1.66E + 06	1.63E + 06 ± 1.36E + 05	0.15^A^	0.38^A^	0.064937
	0.000002	0.933157		0.000001	0.000244	*p* value

^a,b,c^Different lowercase letters on the superscript of values indicate significance difference due to “time category” of MANOVA among a row by Tukey post hoc test (*p* < 0.05). ^A,B,C^Different uppercase letters on the superscript of values indicate significance difference due to “substrate category” of MANOVA among a column by Tukey post hoc test (*p* < 0.05); *n.a.*, not analyzed; *n.d.*, 0; *BL*, baseline of colonic fermentation; *SD*, standard deviation; *T1*, 18 h; *EP*, 24 h

### Metabolomics

#### Discrimination of the volatilome of different samples

**Fig. 1:**
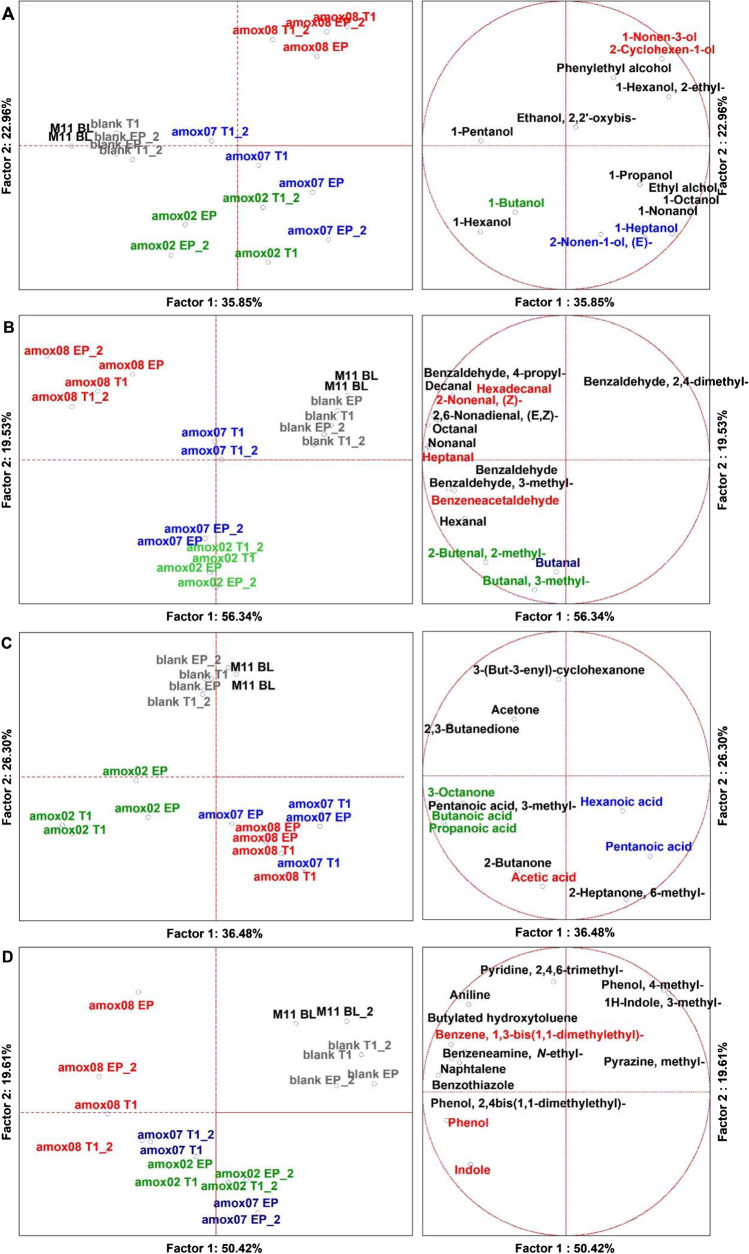
PCA of the volatilome of colonic fermentation samples. **A** alcohols; **B** aldehydes; **C** organic acids and ketones; **D** aromatic compounds. M11 BL, baseline (2.10 h); T1, intermediate time point (18 h); EP, endpoint (24 h). Different colors on variables indicates respective descriptors by MANOVA (*p* < 0.05) (Supplemental Table S3 and S4)

Through SPME GC–MS, among 18 duplicated cases (*n* = 36), 158 molecules were identified with more than 80% of similarity with NIST 11 MSMS library and the NIST MS Search program 2.0 (NIST, Gaithersburg, MD, USA). On average, 96 were relatively quantified at the BL, while 137 were quantified during the 24 h of experiments at different timepoints. For a landscape description of the volatilome, a dataset of 56 significant molecules (ANOVA *p* < 0.05) was generated, then sorted and super-normalized by similar chemical classes of VOCs, i.e., aldehydes, alcohols, acids and ketones, and other aromatics (alkanes were excluded) (Nissen et al. 2020). In details, within the 17 aldehydes quantified, 6 were found at the BL, 16, 17, and 16 were found during fermentation of amox02, amox07, and amox08, respectively. Within the 14 alcohols quantified, 9 were found at the BL, 13, 14, and 12 were found during fermentation of amox02, amox07, and amox08, respectively. Within the 6 organic acids quantified, 3 were found at the BL, 5, 5, and 3 were found during fermentation of amox02, amox07, and amox08, respectively. Within the 6 ketones quantified, 4 were found at the BL, 6, 5, and 4 were found during fermentation of amox02, amox07, and amox08, respectively. From each dataset, multivariate analyses, such as untargeted principal component analysis (PCA) (Fig. 1) and targeted MANOVA (*p* < 0.01) (Supplemental Table S3 and S4) were achieved to address the specific contributes to VOCs production by the independent variables. Super-normalization of the dataset was essential to unveil the effect of those compounds that are less volatile than others and could be underrepresented, as well as to avoid comparing one chemical class to another.

A PCA of 14 statistically significant alcohols has distributed cases on the plot, discriminating the BL (M11 BL) variables to the fermentation of any milk samples, but not to that of the BC (Fig. 1A). From our results, the main descriptors of fermentation with the milk samples were 1-butanol for amox02, 1-heptanol and 2-nonen-1-ol, (*E*) for amox07, and 1-nonen-3-ol and 2-cyclohexen-1-ol for amox08 (MANOVA *p* < 0.01). Considering the effect of time on the production of these VOCs, the major contributions were derived from the EPs (MANOVA *p* < 0.01).

A PCA of 16 statistically significant aldehydes showed distributed cases on the plot, separating the BL from the fermentations of milk samples, but not from the blank control (Fig. 1B). From our results, the main descriptors of fermentation with the milk samples were 2-butenal, 2-methyl and butanal, 3-methyl for amox02, butanal for amox07, and hexadecanal and 2-nonenal, (*Z*) for amox08 (MANOVA *p* < 0.01). Considering the effect of time on the production of these VOCs, the major contribution for 2-nonenal, (*Z*) was derived from T1 (18 h) and for the others the major contribution was derived from the EPs. (MANOVA *p* < 0.01).

A PCA of 12 statistically significant ketones and organic acids distributed cases on the plot, separating the substrates from each other and from the BL, except for the blank control (Fig. 1C). Descriptors of fermentation were butanoic, propanoic acids, and 2-octanone for amox02, pentanoic and hexanoic acids for amox07, and acetic acid for amox08.

A PCA of 13 statistically significant aromatic VOCs distributed cases on the plot, separating the substrates from each other and from the BL, but not form the BC (Fig. 1D). Otherwise, considering the MANOVA, the main descriptors of fermentation were mainly addressed to amox08 cases. In particular, principal descriptors of this sample fermentation were indole and phenol.

So far, the volatilome of colonic fermentation of mother’s milk containing antibiotic residues was described by positive features, such as higher acetic acid, but also by negative ones, such as the higher indole and phenol loads.

#### Shift of beneficial or detrimental microbial metabolic indicators

**Fig. 2:**
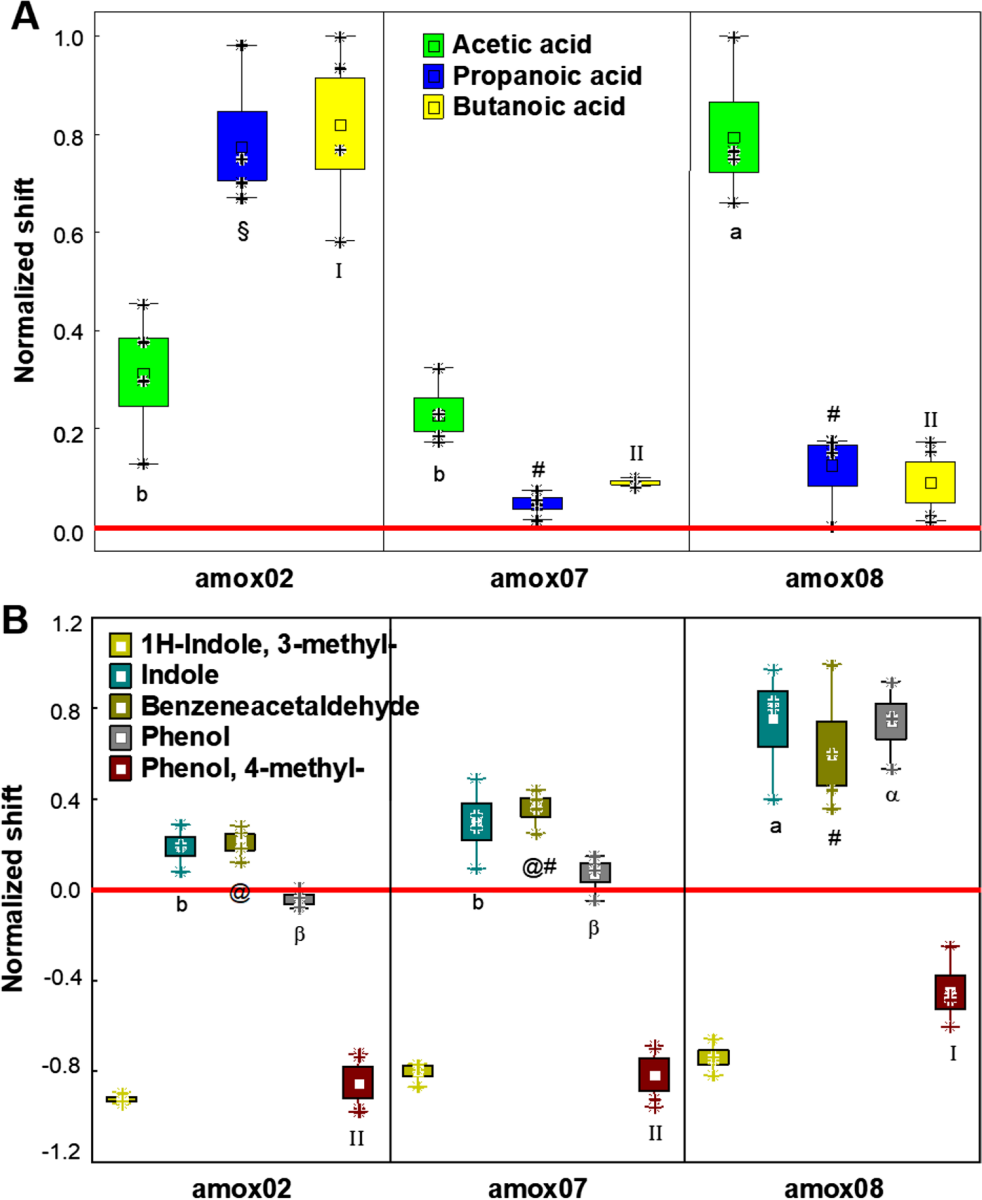
Changes in the abundance of **A** beneficial microbial VOCs metabolites and of **B** detrimental VOCs metabolites, expressed as normalized scale from relative abundances with respect to the baseline (red line). The baseline absolute quantifications in mg/kg are found in the Supplementary Material (Supplemental Table S2). Changes were recorded after 18, and 24 h of in vitro fecal batch fermentations with amox02, amox07, amox08, and a blank control. Each plot is made with the raw data obtained from each time point and replica. Samples were analyzed in duplicate from two independent experiments (*n* = 4). Marker, mean; box, mean ± S.D.; whiskers, min–max; asterisks, raw data. Cases with different letters or numbers or symbols among a single independent variable are significantly different according to Tukey’s HSD test (*p* < 0.05)

To analyze the production of principal volatile microbial metabolites related to food fermentations, we have considered the quantity differences from the BL to the EP, including T1 of eight selected VOCs (ANOVA *p* < 0.05) with renowned bioactivity in humans (short and medium chain organic acids and aromatic compounds). In this elaboration of results, we chose not to include the case of the blank control, because the output generated by volatilome analyses found no discrimination for this case. The first group of VOCs is relative to low organic acids, such as acetic, propanoic, and butanoic acid, that are beneficial compounds essential for the piglets’ gut mucosa and the eubiosis of the colon microbiota (Fig. 2A). The second set is relative to compounds related to proteolytic fermentation and/or detrimental for the piglet’s gut mucosa, such as indole, 1H-indole, 3-methyl (skatole), phenol, phenol, 4-methyl (*p*-cresol), and benzeneacetaldehyde (Fig. 2B).

Results shown in Fig. 2A indicate that acetic, propanoic, and butanoic acids concentration was increased from small amounts detected at the BL (Supplemental Table S2), with any milk sample. Specifically, amox02 fermentation produced the top amounts of propanoic and butanoic acid, but little quantity of acetic acid. In contrast, the amox08 fermentation produced the top amount of acetic acid, but little quantity of propanoic and butanoic acid.

Results shown in Fig. 2B indicate that starting from BL values (Supplemental Table S2) detrimental aromatic VOCs concentration trended similarly during any milk sample fermentation. skatole and *p*-cresol were reduced, while indole, phenol, and benzeneacetaldehyde were increased, after colonic fermentation in respect to the BL. In particular, there were significative differences between amox02 and amox08 in the production of indole and benzeneacetaldehyde and in the reduction of *p*-cresol. In particular, the former two were produced 3.9-folds more and 2.8-fold more in amox08 than amox02, respectively.

## Discussion

It was reported that early-life in-feed antibiotic treatments could alter the gut microbiota of young piglets, affecting digestive physiology, with greater respect to carbohydrates metabolism (Mu et al. 2017; Lin et al. 2018) and future growth (Yu et al. 2018). Indeed, once ingested, amoxicillin undergoes acid-catalyzed degradation in the stomach and enzymatic degradation by intestinal flora; previous studies showed the presence of various beta-lactamase enzymes in the normal intestinal microbiota of juvenile pigs (Reyns et al. 2008). Being amoxicillin a hydrophilic drug, it can be mainly found in the liquid fraction once ingested through milk (Ozdemir et al. 2018); however, using a gut model adapted to suckling piglets’ colon microbiota represents a valuable approach to study gut microbiota shift and their metabolites as a consequence of milk amoxicillin residues absorption.

### Microbiomics

#### Biodiversity of colonic microbiota

From the alpha diversity analyses, the resulting scenario indicates that generally the eubiosis in respect to the BL was maintained after colonic milk fermentations by any index, with the exception for significant reductions in evenness. This effect commonly happens in the in vitro colon models, because underrepresented taxa use to grow slower than the core microbiota, disrupting the evenness of distribution. The Good’s index that had no significative differences confirms the stability of MICODE environment throughout fermentation. Beta diversity indicated that the shifts happened in the microbiota of piglets after milk fermentation were not so dramatic and overall the differences among samples from colonic fermentations were limited.

#### Relative and absolute quantification of colonic microbiota and milk microflora

By the quantifications reported by qPCR analyses, overall, in milk samples the presence of amoxicillin resulted in lower bacterial loads, that desirably were relative to reduction of opportunistic bacteria, but also and undesirably to commensals *Lactobacillales* and to depletion of *Bifidobacteriaceae*. These loads of exogenous microbes should not have impacted on the colon microbiota, because are at least 1,000,000 times lower than what was quantified at the baseline of fermentation.

For the intestinal health of piglets, the capability to reduce the content of opportunistic and pathogens, such as those included in the families *Staphylococcaceae*, *Enterobacteriaceae*, and *Desulfovibrionaceae*, is an important goal, because these bacterial taxa are culprits of dysbiosis induction and can led to intestinal pathogenesis (Gresse et al. 2017; Hasan et al. 2018). For example, the first family is generally transferred to the piglets’ colon from the batch flora of the mammary glands and some species are associated with several important piglets’ pathologies (Wang et al. 2017).

For the intestinal health of piglets, the *Lactobacillales* order is fundamental. Since the first days, the piglets’ colon microbiota is dominated by *Lactobacillaceae* mainly, accounting for a third of the whole pie (Petri et al. 2010). This taxon is inherited from the sow milk, that in our sow milk samples had a mean value of 2.01E + 05 cells/mL, and establish an important symbiosis up to the adult phase, contributing to the microbiota’s beneficial effects (Petri et al. 2010). Additionally, several lactobacilli strains of pig’s origin were proposed as probiotic and porcine feed additive, e.g., *Lactobacillus salivarius* LS6 (Yeo et al. 2016). However, in our work, this community was reduced by the action of antibiotic residue in milk.

The inhibitory activity against commensals and beneficial taxa is clearly a side effect of wide range antibiotics, such as amoxicillin, that other than the opportunistic taxa also reduce largely the richness of the microbiota, including the split in beneficial bacteria.

For the intestinal health of piglets, the role of *Clostridiales* is crucial, because represents a large portion of the core microbiota, accounting generally for the 30% of the colon microbiota of piglets. The reduction of *C. perfringens*, *C. baratii*, and *C. frigidicarnis* is fine since are causative agents of enteritis in pigs and used to spread in herds, additionally toxigenic *C. perfringens* can lead to death, and also represent a risk for the consumers (Mehdizadeh Gohari et al. 2021). Also, the increased abundance of *C. butyricum* has to be observed as a positive feature, since this taxon is a butyrate producer and has been proposed and successfully tested as a probiotic for weanling pigs feed (Peeters et al. 2019; Casas et al. 2020).

Considering the overall scenario, there is evidence that some amoxicillin resistant taxa took advantage of the depletion of abundant opportunistic sensitive ones. For example, three are the cases encountered in our work: (i) the split in the *Lactobacillales* class, where the *Enterococcaceae* took advantage from the depletion of *Lactobacillaceae*. Some species of *Enterococcaceae* have been recently used as probiotics for post-weaning pigs (Sato et al. 2019). In contrast, in poultry some species can cause bacteremia, especially during antibiotic treatment, because are reported to be resistant to amoxicillin (Cuccato et al. 2021). (ii) The split in the *Clostridiales* class, which deepened at the lowest taxonomic level, was driven by amox08 fermentation. This taxon was reduced for its overall content, but the reduction was higher for the portion of the more sensitive taxa than that of the tougher taxa. Among these former taxa, there are also some reported to be generally resistant to antibiotic and also specifically to amoxicillin as follows: *Peptostreptococcaceae* and the *Clostridiaceae* (de Jong et al. 2014). (iii) The split in *Gammaproteobacteria* order is described by the constrained growth of *Enterobacteriaceae* and the rise in the abundance of *Pasteurellaceae.* Even in this situation, the reduction of *Enterobacteriaceae* from our results is a positive feature to maintain a healthy colon of the animals, but *Pasteurellaceae* are important pathogens affecting the respiratory tract of pigs, which in the past were susceptible to antibiotic treatments (de Jong et al. 2014), but nowadays are becoming resistant developing specific phenotypes (Gao et al. 2021).

### Metabolomics

#### Volatilome

The results that we have presented have highlighted that in respect to the BL, there were no fermentation differences for the BC, but there were discriminations in respect to the milk fermentations, and that each one had typical descriptors mainly produced at the EP. This means that the fermentations of milk substrates produced different profiles of VOCs, because made a different impact on the colon microbiota.

For example, among alcohols the production of 1-butanol described the milk with no antibiotics, while 2-cyclohexen-1-ol that of amox08. The colon microbiota produces different alcohols during fermentation of dietary polysaccharides. For example, 1-butanol is a product of butanoic acid fermentation that happens when the pH is not low enough to ensure the exclusive activity of lactic acid bacteria, as should happen in a healthy piglet colon, maybe due to the action of clostridia. In fact, it is reported that *Clostridium acetobutylicum* produces less acids and more neutral products like butanol, thus carrying out acetone butanol fermentation (Ciani et al. 2013). 2-Cyclohexen-1-ol was probably produced from microbial transformation of amoxicillin building blocks, like cyclohexenone (Jiang et al. 2020).

Among aldehydes, benzeneacetaldheyde and 2-butenal, 2-methyl were found to be descriptors of fermentation of milks with and without antibiotic residues, respectively. The aldehydes that are a result of microbial fermentation of lipids could be health-promoters, like 2-butenal, 2-methyl that was reported to limit the growth of several intestinal pathogens at a very low concentration (Zhang et al. 2020) and could have contributed to the management of a natural occurring eubiosis of colon microbiota. Also, other aldehydes are detrimental, being cytotoxic at a low threshold, such as benzeneacetaldheyde (Zhang et al. 2020), that in our work could have been derived from bacterial fermentation of phenylalanine, that is typically rich in milk proteins. The higher amount of this aldehyde found after fermentation of milk with antibiotic residues could have been produced by the higher abundance of *E. faecalis* that characterized the end of amox08 fermentation. In fact, this taxon is known for its selectivity in fermentation of phenylalanine in respect to lactobacilli (Canon et al. 2021).

Other descriptors of the volatilome that discriminated the fermentation with and without antibiotic residues were the organic acids, with acetic acid for the amox08 and butanoic and propanoic acids for amox02, and also indoles that described principally amox08. These compounds will be discussed later.

#### Dysbiosis metabolite indicators

A reduction in acetic, propanoic, and butanoic acids abundances is linked to dysbiosis of the colon microbiota and a reduced intestinal cell homeostasis (Gibson et al. 2017). Thus, from our results, no sample was able to disrupt the proper colonic fermentation of milk, because the three of them increased the production of these VOCs in respect to the BL. The different scenario observed in the production of low organic acids could be principally addressed to the increased abundance of enterococci to the reduction of lactobacilli for the production of acetic acid, and to the reduction of butyrate producers bacteria (e.g., *Ruminococaceae* and *Lachnospiraceae*) seen in amox08.

Enterococci have pyruvate dissimilation that follows several pathways leading to at least five fermentation end-products including acetate (Snoep et al. 1991). In line with our results, Fujita et al. (2020) reported that the supplementation of pigs fed with *Enterococcus faecium* increased fecal acetate levels, which plays an important role for maintaining immune functions. Oppositely, the reduced microbial production of butanoic acid seen in amox08 in respect to amox02 has to be linked to the undesired inhibitory effect of the antibiotic residues towards sensible commensal *Clostridiales* that are butyrate producers. In particular, we have observed a reduction in *Roseburia* and *F. prausnitzii*. Butanoic acid in piglets is produced mainly by the colon microbiota and is a preferential nutrient for energy production by the colonocyte (Kien et al. 2002). Also, it is an important modulator of gut cellular homeostasis, and when it is administered in diet as sodium butyrate alleviates diarrhea symptoms and decreased intestinal permeability without affecting the growth of early weaned piglets (Feng et al. 2018).

In pigs, skatole and indole are formed by the microbial degradation of L-tryptophan in the large intestine and contribute to the typical development of boar taint (Witte et al. 2021). L-Tryptophan accumulates especially in the colon when protein sources are used with a low pre-cecal digestibility (Leong et al. 2011).

The reduction of these compounds is due to the liver, but when their concentrations is excessive can accumulate also in the adipose tissue (Witte et al. 2021), resulting in a commercial loss. From our results, the higher increase in indole of amox08 in respect to amox02 could be due to the reduced abundance of *Lactobacillaceae* observed in the presence of antibiotic residues. In fact, this taxon in the colon can transform indole into beneficial compounds (e.g., indole propionic acid) (Konopelski and Mogilnicka 2022).

Similarly to indoles, phenol and *p*-cresol are derived from proteolytic fermentation of undigested or partially digested proteins and have been shown to damage the gut mucosa disrupting the epithelial barrier function and being genotoxic (Al Hinai et al. 2019; Wang et al. 2020). Also in farm animals, the excessive production of these metabolites can affect the quality of meat and milk and is a source of contaminating emissions from animal manure (Gasaly and Gotteland 2022). In our work, phenol and *p*-cresol should be derived from fermentation of tyrosine due to proteolysis of milk. From our results, the capacity of amox08 fermentation to reduce less the amount of *p*-cresol than what the control milk did could still be attributed to a lower content in *Lactoabacillaceae*, as it has been reported in a similar in vitro colon model, where the correlation among lactobacilli and *p*-cresol was negative (Al Hinai et al. 2019).

### Swine model

Within antibiotics for use in animals, the European Medicines Agency (EMA 2020) has currently classed amoxicillin, without beta-lactamase inhibitors, as category D antibiotic; therefore, it is highly recommended as first line treatment and, as always, should be used prudently only when medically needed (EMA/CVMP/CHMP/682198/2017). The establishment of the intestinal microbiota is a pivotal step in newborn piglets; thus, the effects of antibiotics such amoxicillin in early-life stages could critically affect gut microbial development and future growth (Mu et al. 2017; Lin et al. 2018; Yu et al. 2018). This statement is especially true amid zootechnical industry, where intensive farming pigs undergo fast and massive weight increment. The present in vitro experiment using an innovative colon model allowed authors to carry out a preliminary study avoiding any unnecessary harm to the piglets and the sows as well, still obtaining reliable data on microbial shift due to amoxicillin residues in sows’ milk.

Digestive enzyme secretory patterns seem to be of relevance in the process of assimilation of milk components; indeed, previous studies showed that maturation of gastric, pancreatic, and biliary digestive fluids occurs at an early period of life, starting gradual maturation around the sixth day of life (Manners 1976; Corring et al. 1978; Harada et al. 1988). To the authors’ knowledge, there are yet no researches that evaluated amoxicillin digestion and absorption in newborn piglets. In this study, 7 days old piglets’ fecal samples to build up the in vitro colon microbiota model were used; therefore, as the newborn had an immature digestive capacity, the milk samples were directly fermented in the colon model with no gastric phase digestion.

In conclusion, the early establishment of a stable gut microbiota is pivotal for the pigs’ gastrointestinal physiological functions, also affecting future growth performance and therefore, investigating exogenous molecules effects on these indigenous microbes is of great importance in swine production. In this work, the pig model was adopted to study the role of sow’s milk in modifying antibiotic resistant gut microbiota for the first time in combination to a gut model. Moreover, a wider understanding was allowed by a metabolomic approach. The use of MICODE, a robust and versatile in vitro model, together with multivariate statistics visibly demonstrated a suitable approach to describe the effects generated by milk containing amoxicillin residues towards the colon microbiota of suckling piglets. To fully understand the transfer of antibiotic from sow’s milk to the piglets, in vivo trials are imperative; however, the results presented are target-effective and should be reliable for preclinical investigations. Due to the results obtained, this experimental approach looks suitable to study some mechanisms of antibiotic resistance transfer as well. Furthermore, such in vitro approach could be included in a pipeline of experiments reducing the number of living animals testing, according to the Directive 2010/63/EU and the Regulation (EU) 2019/1010.

## Supplementary Information

Below is the link to the electronic supplementary material.

Supplementary file1 (PDF 545 KB)

## Data Availability

Data are available upon reasonable request to the corresponding author. The NCBI Bioproject PRJNA862673 is available at https://www.ncbi.nlm.nih.gov/bioproject/862673, including Biosamples and relative SRAs, that will be release at least 2022–12-15, or with the release of linked data, whichever is first.

## References

[CR1] Al Hinai EA, Kullamethee P, Rowland IR, Swann J, Walton GE, Commane DM (2019). Modelling the role of microbial p-cresol in colorectal genotoxicity. Gut Microbes.

[CR2] Asare PT, Greppi A, Pennacchia A, Brenig K, Geirnaert A, Schwab C, Stephan R, Lacroix C (2021) In vitro modeling of chicken cecal microbiota ecology and metabolism using the PolyFermS platform. Front Microbiol 12:780092 10.3389/fmicb.2021.780092 PMC8721126 34987487

[CR3] Bonfrate L, Di Palo DM, Celano G, Albert A, Vitellio P, De Angelis M, Gobbetti M, Portincasa P (2020) Effects of Bifidobacterium longum BB536 and *Lactobacillus rhamnosus* HN001 in IBS patients. Eur J Clin Investig 50(3):e13201 10.1111/eci.13201 31960952

[CR4] Burch DGS, Sperling D (2018). Amoxicillin—current use in swine medicine. J Vet Pharmacol Ther.

[CR5] Canon F, Maillard M-B, Henry G, Thierry A, Gagnaire V (2021). Positive interactions between lactic acid bacteria promoted by nitrogen-based nutritional dependencies. Appl Environ Microbiol.

[CR6] Caporaso JG, Kuczynski J, Stombaugh J, Bittinger K, Bushman FD, Costello EK, Fierer N, Peña AG, Goodrich JK, Gordon JI (2010). QIIME allows analysis of high-throughput community sequencing data. Nat Methods.

[CR7] Casas GA, Blavi L, Cross T-WL, Lee AH, Swanson KS, Stein HH (2020). Inclusion of the direct-fed microbial *Clostridium butyricum* in diets for weanling pigs increases growth performance and tends to increase villus height and crypt depth, but does not change intestinal microbial abundance. J Anim Sci.

[CR8] Casciano F, Nissen L, Gianotti A (2021). Effect of formulations and fermentation processes on volatile organic compounds and prebiotic potential of gluten-free bread fortified by spirulina (*Arthrospira platensis)*. Food Funct.

[CR9] Ciani M, Comitini F, Mannazzu I, Fath B (2013). Fermentation☆. Encyclopedia of ecology.

[CR10] ConcePTION (n.d.). https://www.imi-conception.eu/background/description/. Accessed 30 Jun 2022

[CR11] Corring T, Aumaitre A, Durand G (1978). Development of digestive enzymes in the piglet from birth to 8 weeks. Ann Nutr Metab.

[CR12] Cuccato M, Rubiola S, Giannuzzi D, Grego E, Pregel P, Divari S, Cannizzo FT (2021). 16S rRNA sequencing analysis of the gut microbiota in broiler chickens prophylactically administered with antimicrobial agents. Antibiotics.

[CR13] de Jong A, Thomas V, Simjee S, Moyaert H, El Garch F, Maher K, Morrissey I, Butty P, Klein U, Marion H (2014). Antimicrobial susceptibility monitoring of respiratory tract pathogens isolated from diseased cattle and pigs across Europe: the VetPath study. Vet Microbiol.

[CR14] Di Cagno R, De Angelis M, De Pasquale I, Ndagijimana M, Vernocchi P, Ricciuti P, Gagliardi F, Laghi L, Crecchio C, Guerzoni ME (2011). Duodenal and faecal microbiota of celiac children: molecular, phenotype and metabolome characterization. BMC Microbiol.

[CR15] Edgar RC (2010). Search and clustering orders of magnitude faster than BLAST. Bioinformatics.

[CR16] EMA (2020) Categorisation of antibiotics used in animals promotes responsible use to protect public and animal health. In: Eur Med Agency. https://www.ema.europa.eu/en/news/categorisation-antibiotics-used-animals-promotes-responsible-use-protect-public-animal-health. Accessed 30 Jun 2022

[CR17] Feng W, Wu Y, Chen G, Fu S, Li B, Huang B, Wang D, Wang W, Liu J (2018). Sodium butyrate attenuates diarrhea in weaned piglets and promotes tight junction protein expression in colon in a GPR109A-dependent manner. Cell Physiol Biochem.

[CR18] Fujita S, Baba Y, Nakashima Y, HigashimuraY YK, Matsuzaki C, Kawagishi M (2020). Administration od *Enterococcus faecium* HS-08 increases intestinal acetate and induces immunoglobulin A secretion in mice. Can J Microbiol.

[CR19] Gao Y, Xia L, Pan R, Xuan H, Guo H, Song Q, Wei J, Shao D, Liu K, Li Z (2021). Identification of *mcr-1* and a novel chloramphenicol resistance gene *catT* on an integrative and conjugative element in an *Actinobacillus* strain of swine origin. Vet Microbiol.

[CR20] Gasaly N, Gotteland M (2022). Interference of dietary polyphenols with potentially toxic amino acid metabolites derived from the colonic microbiota. Amino Acids.

[CR21] Gibson GR, Hutkins R, Sanders ME, Prescott SL, Reimer RA, Salminen SJ, Scott K, Stanton C, Swanson KS, Cani PD (2017). Expert consensus document: the International Scientific Association for Probiotics and Prebiotics (ISAPP) consensus statement on the definition and scope of prebiotics. Nat Rev Gastroenterol Hepatol.

[CR22] Gresse R, Chaucheyras-Durand F, Fleury MA, Van de Wiele T, Forano E, Blanquet-Diot S (2017). Gut microbiota dysbiosis in postweaning piglets: understanding the keys to health. Trends Microbiol.

[CR23] Guerzoni ME, Vernocchi P, Ndagijimana M, Gianotti A, Lanciotti R (2007). Generation of aroma compounds in sourdough: effects of stress exposure and lactobacilli–yeasts interactions. Food Microbiol.

[CR24] Harada E, Kiriyama H, Kobayashi E, Tsuchita H (1988). Postnatal development of biliary and pancreatic exocrine secretion in piglets. Comp Biochem Physiol A.

[CR25] Hasan S, Junnikkala S, Peltoniemi O, Paulin L, Lyyski A, Vuorenmaa J, Oliviero C (2018). Dietary supplementation with yeast hydrolysate in pregnancy influences colostrum yield and gut microbiota of sows and piglets after birth. PLoS ONE.

[CR26] Isaacson R, Kim HB (2012). The intestinal microbiome of the pig. Anim Health Res Rev.

[CR27] Isenring J, Geirnaert A, Lacroix C, Stevens MJ (2021). Bistable auto-aggregation phenotype in *Lactiplantibacillus plantarum* emerges after cultivation in in vitro colonic microbiota. BMC Microbiol.

[CR28] Jiang H, Qu Z, Liu Y, Liu X, Wang G, Wang Y, Xu L, Ding K, Xing W, Chen R (2020). Pilot-scale cyclohexanone production through phenol hydrogenation over Pd/CN in a continuous ceramic membrane reactor. Ind Eng Chem Res.

[CR29] Kien CL, Chang JC, Cooper JR (2002). Quantitation of colonic luminal synthesis of butyric acid in piglets. J Pediatr Gastroenterol Nutr.

[CR30] Konopelski P, Mogilnicka I (2022). Biological effects of indole-3-propionic acid, a gut microbiota-derived metabolite, and its precursor tryptophan in mammals’ health and disease. Int J Mol Sci.

[CR31] Koutsos A, Lima M, Conterno L, Gasperotti M, Bianchi M, Fava F, Vrhovsek U, Lovegrove JA, Tuohy KM (2017). Effects of commercial apple varieties on human gut microbiota composition and metabolic output using an in vitro colonic model. Nutrients.

[CR32] Leong J, Morel PC, Purchas RW, Wilkinson BH (2011). Effects of dietary components including garlic on concentrations of skatole and indole in subcutaneous fat of female pigs. Meat Sci.

[CR33] Lin C, Wan J, Su Y, Zhu W (2018). Effects of early intervention with maternal fecal microbiota and antibiotics on the gut microbiota and metabolite profiles of piglets. Metabolites.

[CR34] Love MI, Huber W, Anders S (2014). Moderated estimation of fold change and dispersion for RNA-seq data with DESeq2. Genome Biol.

[CR35] Luo Y, Ren W, Smidt H, Wright A-DG, Yu B, Schyns G, McCormack UM, Cowieson AJ, Yu J, He J (2022) Dynamic distribution of gut microbiota in pigs at different growth stages: composition and contribution. Microbiol Spectr e00688–21 10.1128/spectrum.00688-21 PMC9241710 35583332

[CR36] Manners MJ (1976). The development of digestive function in the pig. Proc Nutr Soc.

[CR37] Marino M, de Wittenau GD, Saccà E, Cattonaro F, Spadotto A, Innocente N, Radovic S, Piasentier E, Marroni F (2019). Metagenomic profiles of different types of Italian high-moisture Mozzarella cheese. Food Microbiol.

[CR38] McDonald D, Price MN, Goodrich J, Nawrocki EP, DeSantis TZ, Probst A, Andersen GL, Knight R, Hugenholtz P (2012). An improved Greengenes taxonomy with explicit ranks for ecological and evolutionary analyses of bacteria and archaea. ISME J.

[CR39] Mehdizadeh Gohari I, Navarro MA, Li J, Shrestha A, Uzal F, McClane BA (2021). Pathogenicity and virulence of *Clostridium perfringens*. Virulence.

[CR40] Modesto M, Stefanini I, D’Aimmo MR, Nissen L, Tabanelli D, Mazzoni M, Bosi P, Strozzi GP, Biavati B (2011). Strategies to augment non-immune system based defence mechanisms against gastrointestinal diseases in pigs. NJAS-Wagening J Life Sci.

[CR41] Mu C, Yang Y, Su Y, Zoetendal EG, Zhu W (2017). Differences in microbiota membership along the gastrointestinal tract of piglets and their differential alterations following an early-life antibiotic intervention. Front Microbiol.

[CR42] Nauwelaerts N, Deferm N, Smits A, Bernardini C, Lammens B, Gandia P, Panchaud A, Nordeng H, Bacci ML, Forni M (2021). A comprehensive review on non-clinical methods to study transfer of medication into breast milk–a contribution from the ConcePTION project. Biomed Pharmacother.

[CR43] Nissen L, Rollini M, Picozzi C, Musatti A, Foschino R, Gianotti A (2020). Yeast-free doughs by *Zymomonas mobilis*: evaluation of technological and fermentation performances by using a metabolomic approach. Microorganisms.

[CR44] Nissen L, Casciano F, Chiarello E, Di Nunzio M, Bordoni A, Gianotti A (2021). Colonic in vitro model assessment of the prebiotic potential of bread fortified with polyphenols rich olive fiber. Nutrients.

[CR45] Nissen L, Valerii MC, Spisni E, Casciano F, Gianotti A (2021). Multiunit in vitro colon model for the evaluation of prebiotic potential of a fiber plus D-limonene food supplement. Foods.

[CR46] Ozdemir Z, Tras B, Uney K (2018). Distribution of hydrophilic and lipophilic antibacterial drugs in skim milk, cream, and casein. J Dairy Sci.

[CR47] Peeters L, Mostin L, Wattiau P, Boyen F, Dewulf J, Maes D (2019). Efficacy of *Clostridium butyricum* as probiotic feed additive against experimental *Salmonella* Typhimurium infection in pigs. Livest Sci.

[CR48] Petri D, Hill JE, Van Kessel AG (2010). Microbial succession in the gastrointestinal tract (GIT) of the preweaned pig. Livest Sci.

[CR49] Reyns T, De Boever S, De Baere S, De Backer P, Croubels S (2008). Tissue depletion of amoxicillin and its major metabolites in pigs: influence of the administration route and the simultaneous dosage of clavulanic acid. J Agric Food Chem.

[CR50] Sato Y, Kuroki Y, Oka K, Takahashi M, Rao S, Sukegawa S, Fujimura T (2019). Effects of dietary supplementation with *Enterococcus faecium* and *Clostridium butyricum*, either alone or in combination, on growth and fecal microbiota composition of post-weaning pigs at a commercial farm. Front Vet Sci.

[CR51] Shennan DB, Peaker M (2000). Transport of milk constituents by the mammary gland. Physiol Rev.

[CR52] Snoep JL, Joost M, de Mattos T, Neijssel OM (1991). Effect of the energy source on the NADH/NAD ratio and on pyruvate catabolism in anaerobic chemostat cultures of *Enterococcus faecalis* NCTC 775. FEMS Microbiol Lett.

[CR53] Tamargo A, Cueva C, Silva M, Molinero N, Miralles B, Bartolomé B, Moreno-Arribas MV (2022). Gastrointestinal co-digestion of wine polyphenols with glucose/whey proteins affects their bioaccessibility and impact on colonic microbiota. Food Res Int.

[CR54] Tanner SA, Zihler Berner A, Rigozzi E, Grattepanche F, Chassard C, Lacroix C (2014). In vitro continuous fermentation model (PolyFermS) of the swine proximal colon for simultaneous testing on the same gut microbiota. PLoS ONE.

[CR55] Tsitko I, Wiik-Miettinen F, Mattila O, Rosa-Sibakov N, Seppänen-Laakso T, Maukonen J, Nordlund E, Saarela M (2019). A small in vitro fermentation model for screening the gut microbiota effects of different fiber preparations. Int J Mol Sci.

[CR56] Venardou B, O’Doherty JV, McDonnell MJ, Mukhopadhya A, Kiely C, Ryan MT, Sweeney T (2021). Evaluation of the in vitro effects of the increasing inclusion levels of yeast β-glucan, a casein hydrolysate and its 5 kDa retentate on selected bacterial populations and strains commonly found in the gastrointestinal tract of pigs. Food Funct.

[CR57] Venema K (2015) The TNO in vitro model of the colon (TIM-2). The impact of food bioactives on health, pp 293–304

[CR58] Ventrella D, Forni M, Bacci ML, Annaert P (2019). Non-clinical models to determine drug passage into human breast milk. Curr Pharm Des.

[CR59] Ventrella D, Ashkenazi N, Elmi A, Allegaert K, Aniballi C, DeLise A, Devine PJ, Smits A, Steiner L, Forni M (2021). Animal models for in vivo lactation studies: anatomy, physiology and milk compositions in the most used non-clinical species: a contribution from the ConcePTION project. Animals.

[CR60] Wang Q, Garrity GM, Tiedje JM, Cole JR (2007). Naive Bayesian classifier for rapid assignment of rRNA sequences into the new bacterial taxonomy. Appl Environ Microbiol.

[CR61] Wang M, Hu J, Zhu L, Guo C, Lu H, Guo C, Li X, Wang X (2017). A fatal suppurative pneumonia in piglets caused by a pathogenic coagulase-positive strain of *Staphylococcus hyicus*. Vet Res Commun.

[CR62] Wang X, Gibson GR, Sailer M, Theis S, Rastall RA (2020). Prebiotics inhibit proteolysis by gut bacteria in a host diet-dependent manner: a three-stage continuous in vitro gut model experiment. Appl Environ Microbiol.

[CR63] Westfall S, Lomis N, Prakash S (2018). A novel polyphenolic prebiotic and probiotic formulation have synergistic effects on the gut microbiota influencing *Drosophila melanogaster* physiology. Artif Cells Nanomedicine Biotechnol.

[CR64] Witte F, Pajic A, Menger F, Tomasevic I, Schubert DC, Visscher C, Terjung N (2021). Preliminary test of the reduction capacity for the intestinal adsorption of skatole and indole in weaning piglets by pure and coated charcoal. Animals.

[CR65] Yang Y, Liu Y, Liu J, Wang H, Guo Y, Du M, Cai C, Zhao Y, Lu C, Guo X (2021). Composition of the fecal microbiota of piglets at various growth stages. Front Vet Sci.

[CR66] Yeo S, Lee S, Park H, Shin H, Holzapfel W, Huh CS (2016). Development of putative probiotics as feed additives: validation in a porcine-specific gastrointestinal tract model. Appl Microbiol Biotechnol.

[CR67] Yu M, Mu C, Zhang C, Yang Y, Su Y, Zhu W (2018). Marked response in microbial community and metabolism in the ileum and cecum of suckling piglets after early antibiotics exposure. Front Microbiol.

[CR68] Zhang D, Gong L, Ding S, Tian Y, Jia C, Liu D, Han M, Cheng X, Sun D, Cai P (2020). FRCD: A comprehensive food risk component database with molecular scaffold, chemical diversity, toxicity, and biodegradability analysis. Food Chem.

