# Supplementary material for: Maternal amoxicillin affects piglets colon microbiota: microbial ecology and metabolomics in a gut model
Source: Appl Microbiol Biotechnol. 2022 Oct 14;106(22):7595–614. doi: 10.1007/s00253-022-12223-3 (PMC9666337; doi:10.1007/s00253-022-12223-3)
Supplement: Supplementary file 1 — Supplementary file1 (PDF 545 KB) [file 253_2022_12223_MOESM1_ESM.pdf]

Applied Microbiology and Biotechnology

Maternal amoxicillin affects piglets colon microbiota: microbial ecology and metabolomics in a gut model

Lorenzo Nissen[1,2,§], Camilla Aniballi[3,§], Flavia Casciano[1], Alberto Elmi[3], Domenico Ventrella[3,*], Augusta Zannoni[3,4], Andrea Gianotti[1,2,#], Maria Laura Bacci[3,4,#]

[1] Department of Agricultural and Food Sciences (DISTAL), *Alma Mater Studiorum University of Bologna: Universita di Bologna*, P.za Goidanich 60, 47521, Cesena, Italy.

[2] Interdepartmental Centre of Agri-Food Industrial Research (CIRI-AGRO), *Alma Mater Studiorum University of Bologna: Universita di Bologna*, Via Q. Bucci 336, 47521 Cesena, Italy.

[3] Department of Veterinary Medical Sciences, *Alma Mater Studiorum University of Bologna: Universita di Bologna*, via Tolara di Sopra 50, 40064 Ozzano dell'Emilia (BO), Italy

[4] Health Sciences and Technologies-Interdepartmental Center for Industrial Research (CIRI-SDV), *Alma Mater Studiorum University of Bologna: Universita di Bologna*, 40126 Bologna, Italy

Supplementary Materials

Figure S1, A – F. Plots of Alpha Diversity indices. A = Chao 1 index for microbiota abundance; B = Observed OTU index for microbiota richness; C = Shannon index for microbiota evenness; D = Simpson index for microbiota dominance; E = Good's index for microbiota rarity; F = Bray Curtis PCoA of Beta Diversity. [abc]Different letters indicate significance (ANOVA and Tukey HSD test $p < 0.05$) within a plot.

Table S1. Primers pairs employed for PCR and qPCR reactions and quantifications.

Table S2. Quantification of VOCs by SPME GC/MS related to main microbial VOCs, employing 10000 mg/kg of 2-Penatnol, 4-methyl.

Table S3. Multivariate analyses of the volatilome of different samples at different time points during in vitro colonic fermentation, categorized by "Effect of Time" by MANOVA ($p < 0.05$).

Table S4. Multivariate analyses of the volatilome of different samples at different time points during in vitro colonic fermentation, categorized by "Effect of Substrates" by MANOVA ($p < 0.05$).

Figure S1. Alpha and Beta Biodiversity of colon microbiota after 24 h of fermentation

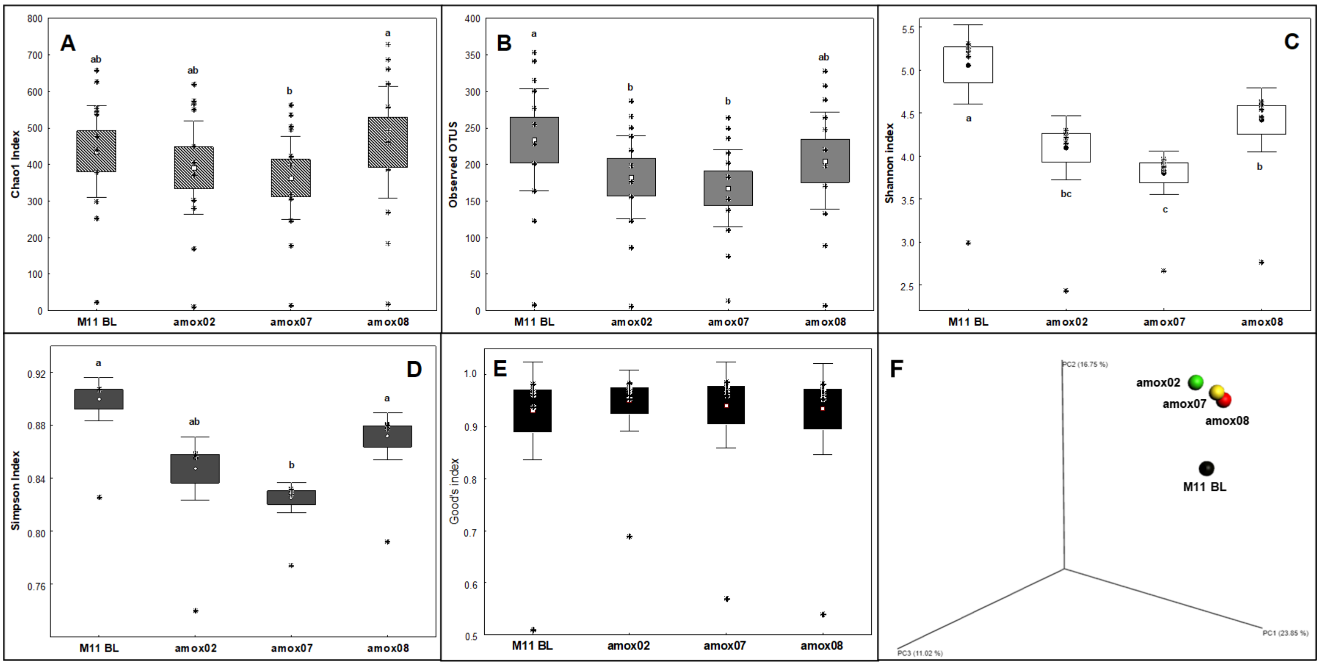

Table S1. Primers pairs employed for PCR and qPCR reactions and quantifications.

**Bacterial taxa**	**Target**	**Sequence 3'-5'**	**Bp***	**MT** (°C)**	**Reference**
Eubacteria	*V3-V4 16S*	Eub518-R: ATTACCGCGGCTGCTGG<br>Eub338-F: ACTCCTACGGGAGGCAG	147	57.6<br>63.5	Lane et al, 1991
Firmicutes	*V3-V4 16S*	Firm-F: GGAGYATGTGGTTTAATT<br>Firm-R: ACTCCTACGGGAGGCAG	300	60.5<br>63.5	Guo et al, 2008
Bacteroidetes	*V3-V4 16S*	Bact-F: GGARCATGTGGTTTAATT<br>Bact-R: AGCTGACGACAACCATG	250	58.9<br>59.4	Guo et al, 2008
Lactobacillales	*V3-V4 16S*	F-Lac: GCAGCAGTAGGGAATCT<br>R-Lac: GCATTYCACCGCTACACA	340	59.8<br>58.3	Walter et al, 2001
Bifidobacteriaceae	*RecA*	RecA-F: CGTYTCBCAGCCGGAYA<br>RecA-R: CCARVGCRCCGGTCATC	220	60.3<br>59.2	Masco et al, 2006
Enterobacteriaceae	*V3-V4 16S*	Enterb-F: TGCCGTAACTTCGGGAG<br>Enterb-R: TCAAGGACCAGTGTTCAG	450	64.2<br>60.3	Bartosh et al, 2004
Clostridium group I	*V3-V4 16S*	ClosI-F: TACCHRAGGAGGAAGCCAC<br>ClosI-R: GTTCTTCCTAATCTCTACGCAT	148	54.6<br>53.0	Bartosh et al, 2004
Clostridium group IV	*V3-V4 16S*	ClosIV-F: TTAACACAATAAGTWATC<br>ClosIV-R: ACCTTCCTCCGTTTTGTC	400	58.1<br>57.9	Goldberg et al, 2013
BPP group	*V3-V4 16S*	BPP-F: GAGAGGAAGGTCCCCCA<br>BPP-R: CGCKACTTGGCTGGTTCA	140	60.5<br>59.9	Pachikian et al., 2011
Escherichia coli	*FtsZ*	EcFtsZ-F: GGTATCCTGACCGTTGCT<br>EcFtsZ-R: ATACCTCGGCCCAGAACT	250	59.4<br>57.3	Zhou & Helmstetter, 1994

*Base pairs, **Melting temperature.

Table S2. Quantification of VOCs by SPME GC/MS related to prebiotic potential, employing 10000 mg/kg of 2-pentanol, 4-methyl.

**VOCs**	**mg/kg ± S.D.**	***p* value**†
Acetic acid	0.031 ± 0.010	0.0013
Propanoic acid	0.019 ± 0.012	0.0469
Butanoic acid	0.061 ± 0.021	0.0231
Indole	25.352 ± 9.795	<0.0001
Phenol	2.912 ± 0.594	<0.0001
Phenol, 4-methyl-	6.193 ± 1.231	0.0156
Benzeneacetaldehyde	0.020 ± 0.007	0.0212
1H-Indole, 3-methyl	0.843 ± 0.043	0.0096

*Traces < 0.01 mg/kg; **phenol, 2,4-bis(1,1-dimethylethyl)-; (LOQ = 0.03 mg/kg and LOD = 0.01 mg/kg); † *p* value of ANOVA from a dataset including all cases and time points.

Table S3. Multivariate analyses of the volatilome of different samples at different time points during in vitro colonic fermentation, categorized by “Effect of Time” by MANOVA ($p < 0.05$).

**VOCs**	**Effect of time to dependent variables production (%)**			**MANOVA**
	BL	T1	EP	*p* value
1-Butanol	37.48	23.73	38.78	0.766684
1-Heptanol	2.49	34.82	62.68	0.410634
1-Hexanol	63.35[a]	18.98[b]	17.66[b]	0.019447
1-Hexanol, 2-ethyl-	0.01	47.40	52.59	0.524728
1-Nonanol	0.01	47.41	52.58	0.089240
1-Nonen-3-ol	0.01	46.82	53.17	0.555965
1-Octanol	0.01[b]	40.04[a]	59.95[a]	0.021290
1-Pentanol	90.12[a]	0.023[b]	9.84[b]	0.000000
1-Propanol	32.73	27.75	39.50	0.477265
2-Nonen-1-ol, (E)-	0.01	72.52	27.47	0.110141
2-Cyclohexen-1-ol	0.01	48.95	51.04	0.145041
Ethyl alchol	5.91[b]	36.11[ab]	57.97[a]	0.038833
Ethanol, 2,2'-oxybis-	14.62[b]	68.73[a]	16.64[b]	0.027514
Phenylethyl alcohol	24.53	24.87	50.58	0.573784
Butanal	0.01	20.23	79.76	0.102151
Butanal, 3-methyl-	0.01	48.17	51.82	0.306084
2-Butenal, 2-methyl-	0.01	49.08	50.91	0.115503
Hexanal	6.90	41.97	51.11	0.147786
Heptanal	0.01	46.17	53.82	0.153683
Octanal	0.01	45.34	54.65	0.126945
Nonanal	0.01	45.60	54.39	0.141468
2-Nonenal, (Z)-	0.01	41.07	58.92	0.311281
Decanal	0.01	48.24	51.75	0.263056
2,6-Nonadienal, (E,Z)-	0.01	50.03	49.96	0.248240
Hexadecanal	0.01	56.94	43.05	0.376285
Benzaldehyde	22.50	42.38	35.10	0.659388
Benzaldehyde, 2,4-dimethyl-	51.20	25.18	23.61	0.084450
Benzaldehyde, 3-methyl-	0.01	59.64	40.35	0.066304
Benzaldehyde, 4-propyl-	27.37	34.12	38.49	0.909027
Benzeneacetaldehyde	2.05	57.81	40.13	0.123854
Benzene, 1,3-bis(dim*)-	26.79	38.14	35.05	0.787677
Benzeneamine, N-ethyl-	32.01	42.15	25.82	0.033509
Benzothiazole	26.58	43.51	29.90	0.153335
Butylated hydroxytoluene	32.02[ab]	41.08[a]	26.88[b]	0.032881
1H-Indole, 3-methyl-	61.81[a]	20.46[b]	17.72[b]	0.029024
Indole	13.95	39.87	46.17	0.224445
Naphtalene	29.10	41.11	29.78	0.378391
Phenol	37.03	24.88	38.07	0.649405
Phenol, 2,4bis(dim*)-	23.74	43.29	32.96	0.299933
Phenol, 4-methyl-	65.96[a]	18.33[b]	15.69[b]	0.036611
Aniline	34.39	40.30	25.30	0.393777

Pyrazine, methyl-	0.01	47.77	52.22	0.310295
Pyridine, 2,4,6-trimethyl-	42.34	26.54	31.11	0.318745

*1,1-dimethylethyl

Table S4. Multivariate analyses of the volatilome of different samples at different time points during in vitro colonic fermentation, categorized by "Effect of Substrates" by MANOVA ($p < 0.05$).

VOCs	Effect of substrates to dependent variables production (%)				MANOVA
	Blank control	amox02	amox07	amox08	*p* value
1-Butanol	28.56[b]	52.87[a]	9.75[c]	8.81[c]	0.037014
1-Heptanol	1.67	30.32	58.25	9.75	0.207862
1-Hexanol	53.54[a]	28.41[b]	18.05[b]	0.00 [c]	0.000187
1-Hexanol, 2-ethyl-	0.01	18.65	22.32	59.03	0.232363
1-Nonanol	0.00[c]	37.77[a]	42.60[a]	19.62[b]	0.033886
1-Nonen-3-ol	0.00[c]	0.00[c]	30.11[b]	69.89[a]	0.020889
1-Octanol	0.00	33.75	40.51	25.73	0.074134
1-Pentanol	85.88[a]	0.38[c]	7.02[b]	6.71[b]	0.000003
1-Propanol	24.49	22.96	28.96	23.57	0.916492
2-Nonen-1-ol, (E)-	0.00[b]	38.30[a]	61.69[a]	0.00[b]	0.006394
2-Cyclohexen-1-ol	0.00[c]	8.67[b]	36.52[a]	54.80[a]	0.000001
Ethyl alchol	4.02	27.27	38.75	29.95	0.193970
Ethanol, 2,2'-oxybis-	10.25	24.15	31.01	34.58	0.800229
Phenylethyl alcohol	17.81	18.41	8.11	55.66	0.135020
Butanal	0.00[b]	42.43[a]	57.56[a]	0.00[b]	0.042168
Butanal, 3-methyl-	0.00[c]	62.24[a]	24.25[b]	13.50[b]	0.000000
2-Butenal, 2-methyl-	0.00[c]	45.84[a]	28.00[b]	26.14[b]	0.000205
Hexanal	4.08[b]	36.62[a]	26.04[a]	33.24[a]	0.030241
Heptanal	0.00[c]	23.48[b]	23.90[b]	52.60[a]	0.000003
Octanal	0.00[c]	23.70[b]	24.57[b]	51.71[a]	0.000000
Nonanal	0.00[c]	24.31[b]	23.68[b]	52.00[a]	0.000003
2-Nonenal, (Z)-	0.00[c]	18.76[b]	16.30[b]	64.93[a]	0.000133
Decanal	0.00[c]	13.88[b]	27.19[b]	58.92[a]	0.000000
2,6-Nonadienal, (E,Z)-	0.00[c]	18.56[b]	22.68[b]	58.75[a]	0.000000
Hexadecanal	0.00[c]	0.00[c]	39.14[b]	60.85[a]	0.000004
Benzaldehyde	15.08	21.95	28.77	34.18	0.699660
Benzaldehyde, 2,4-dimethyl-	45.53	18.81	13.74	21.90	0.002611
Benzaldehyde, 3-methyl-	0.00[c]	30.27[b]	26.72[b]	43.03[a]	0.004375
Benzaldehyde, 4-propyl-	19.15	13.73	19.31	47.79	0.198589
Benzeneacetaldehyde	1.17[c]	29.66[ab]	26.36[b]	42.79[a]	0.027948
2,3-Butanedione	35.63[b]	64.35[a]	0.00[c]	0.00[c]	0.000000
2-Butanone	9.90[b]	34.35[a]	31.76[a]	23.97[a]	0.016575
2-Heptanone, 6-methyl-	0.00[c]	17.42[b]	44.23[a]	38.34[a]	0.000001
3-(But-3-enyl)-cyclohexanone	41.89[a]	19.37[b]	24.83[ab]	13.89[b]	0.038087
3-Octanone	0.00[c]	96.08[a]	3.91[b]	0.00[c]	0.003803
Acetone	32.06	26.84	29.65	11.43	0.077502
Acetic acid	0.00[c]	35.34[a]	16.58[b]	48.06[a]	0.001296
Butanoic acid	0.00[c]	86.61[a]	7.02[b]	6.35[b]	0.017478
Propanoic acid	4.52[c]	72.47[a]	0.00[d]	23.00[b]	0.037235
Pentanoic acid, 3-methyl-	12.49[b]	74.33[a]	13.16[b]	0.00[c]	0.012310
Pentanoic acid	0.00[c]	2.48[c]	66.17[a]	31.33[b]	0.000515

Hexanoic acid	2.48	2.62	84.58	10.31	0.127988
Benzene, 1,3-bis(dim*)-	14.78	37.59	22.68	24.93	0.167975
Benzeneamine, N-ethyl-	19.02	26.05	22.84	32.08	0.450401
Benzothiazole	14.62[b]	27.01[ab]	21.83[ab]	36.53[a]	0.019382
Butylated hydroxytoluene	19.02	29.07	22.26	29.63	0.349089
1H-Indole, 3-methyl-	65.35[a]	10.07[b]	9.87[b]	14.69[b]	0.000000
Indole	6.54[c]	26.17[b]	26.76[b]	40.51[a]	0.000001
Naphtalene	16.57	24.28	30.45	28.68	0.520621
Phenol	23.75[b]	7.04[c]	17.38[bc]	51.81[a]	0.035000
Phenol, 2,4-bis(dim*)-	12.57	30.87	26.62	29.92	0.086084
Phenol, 4-methyl-	78.26[a]	8.24[b]	6.41[b]	7.07[b]	0.000000
Aniline	21.16	33.95	17.97	26.89	0.739773
Pyrazine, methyl-	0.00[b]	36.96[a]	34.82[a]	28.20[a]	0.014902
Pyridine, 2,4,6-trimethyl-	29.65[a]	25.78[ab]	13.04[b]	31.52[a]	0.049508

*1,1-dimethylethyl-
